# Inner Nuclear Membrane Protein, SUN1, is Required for Cytoskeletal Force Generation and Focal Adhesion Maturation

**DOI:** 10.3389/fcell.2022.885859

**Published:** 2022-05-18

**Authors:** Nanami Ueda, Masashi Maekawa, Tsubasa S. Matsui, Shinji Deguchi, Tomoyo Takata, Jun Katahira, Shigeki Higashiyama, Miki Hieda

**Affiliations:** ^1^ Department of Medical Technology, Ehime Prefectural University of Health Sciences, Tobe, Japan; ^2^ Division of Cell Growth and Tumor Regulation, Proteo-Science Center (PROS), Ehime University, Matsuyama, Japan; ^3^ Department of Biochemistry and Molecular Genetics, Ehime University Graduate School of Medicine, Toon, Japan; ^4^ Division of Physiological Chemistry and Metabolism, Graduate School of Pharmaceutical Sciences, Keio University, Tokyo, Japan; ^5^ Division of Bioengineering, Osaka University, Toyonaka, Japan; ^6^ Department of Veterinary Sciences, Osaka Prefecture University, Sakai, Japan; ^7^ Department of Oncogenesis and Growth Regulation, Osaka International Cancer Institute, Osaka, Japan

**Keywords:** LINC complex, SUN proteins, focal adhesion, traction force, actin cytoskeleton, integrin β1

## Abstract

The linker of nucleoskeleton and cytoskeleton (LINC) complex is composed of the inner nuclear membrane-spanning SUN proteins and the outer nuclear membrane-spanning nesprin proteins. The LINC complex physically connects the nucleus and plasma membrane *via* the actin cytoskeleton to perform diverse functions including mechanotransduction from the extracellular environment to the nucleus. Mammalian somatic cells express two principal SUN proteins, namely SUN1 and SUN2. We have previously reported that SUN1, but not SUN2, is essential for directional cell migration; however, the underlying mechanism remains elusive. Because the balance between adhesive force and traction force is critical for cell migration, in the present study, we focused on focal adhesions (FAs) and the actin cytoskeleton. We observed that siRNA-mediated SUN1 depletion did not affect the recruitment of integrin β1, one of the ubiquitously expressed focal adhesion molecules, to the plasma membrane. Consistently, SUN1-depleted cells normally adhered to extracellular matrix proteins, including collagen, fibronectin, laminin, and vitronectin. In contrast, SUN1 depletion reduced the activation of integrin β1. Strikingly, the depletion of SUN1 interfered with the incorporation of vinculin into the focal adhesions, whereas no significant differences in the expression of vinculin were observed between wild-type and SUN1-depleted cells. In addition, SUN1 depletion suppressed the recruitment of zyxin to nascent focal adhesions. These data indicate that SUN1 is involved in the maturation of focal adhesions. Moreover, disruption of the SUN1-containing LINC complex abrogates the actin cytoskeleton and generation of intracellular traction force, despite the presence of SUN2. Thus, a physical link between the nucleus and cytoskeleton through SUN1 is required for the proper organization of actin, thereby suppressing the incorporation of vinculin and zyxin into focal adhesions and the activation of integrin β1, both of which are dependent on traction force. This study provides insights into a previously unappreciated signaling pathway from the nucleus to the cytoskeleton, which is in the opposite direction to the well-known mechanotransduction pathways from the extracellular matrix to the nucleus.

## Introduction

The linker of the nucleoskeleton and cytoskeleton (LINC) complex is a conserved molecular bridge that spans the nuclear envelope and connects the nucleoskeleton and cytoskeleton. The LINC complexes consist of two protein families, namely the Klarsicht, Anc-1, and Syne homology (KASH) domain-containing proteins located on the outer nuclear membrane, and the Sad1 and UNC-84 (SUN) domain-containing proteins embedded in the inner nuclear membrane. The KASH and SUN domains bind to each other in the perinuclear space (Crisp et al., 2006; Razafsky and Hodzic, 2009; Starr, 2011). In the mammalian genome, six genes encode KASH-containing proteins, including four nesprins (nesprin-1–4), KASH5, and the Lymphoid Restricted Membrane protein (LRMP, also called Jaw1), whereas five genes encode SUN-domain-containing proteins, SUN1–5. Nesprins are associated with several cytoskeletal elements in the cytoplasm, including several microtubule motors, filamentous actin (F-actin), and intermediate filaments. Among the four nesprins, nesprin-1 giant (nesprin-1G) and nesprin-2 giant (nesprin-2G) interact with the F-actin in the cytoplasm (Padmakumar et al., 2004) and nesprin-2G is directly shown to be subject to myosin-dependent tension (Arsenovic et al., 2016). Among SUN1–5 proteins, SUN1 and SUN2 are widely expressed in somatic cells, whereas the expression of SUN3, SUN4, and SUN5 is largely restricted to male germ cells (Meinke and Schirmer, 2015). SUN proteins are associated with nuclear lamins and chromatin within the nucleoplasm. LINC complexes can perform diverse and tissue-specific functions, including homeostatic positioning of the nucleus, nuclear migration during development, DNA repair, nuclear shaping, chromosome movements during meiosis, signal transduction, and mechanotransduction (Horn, 2014; Hao and Starr, 2019; Birks and Uzer, 2021; Wong et al., 2021). These functions could be attributed to the variations in the LINC complex components and the availability of a wide range of their binding partners (Hieda, 2017).

Integrins are receptors for the extracellular matrix (ECM), and their clustering induces the formation of nascent focal adhesions (FAs), also known as focal contacts. Certain nascent FAs mature into larger FAs, whereas others are rapidly turned over. FAs contain cytoplasmic scaffolding proteins such as vinculin and paxillin, which are associated with the force-generating actin cytoskeleton. Thus, FAs serve as the mechanical link between the ECM and actin fibers, whose contractility is essential for the maturation of FAs (Gardel et al., 2010). The actin cytoskeleton physically connects with components of the LINC complex, nesprin-1G and nesprin-2G. Accordingly, FAs communicate with the LINC complex *via* the actin cytoskeleton, thereby transmitting several mechanical stimuli originating from outside the cells to the nucleus through the LINC complex (Poh et al., 2012; Versaevel et al., 2012; Lovett et al., 2013; Alam et al., 2015; Cho et al., 2017).

Several studies have indicated that the LINC complex affects cytoskeletal elements and the formation of FAs. For example, perturbation of the LINC complex using dominant-negative KASH (DN-KASH), which broadly interferes with nesprin-SUN interaction, causes impaired propagation of intracellular forces and disturbs the organization of the perinuclear actin and intermediate filament networks (Lombardi et al., 2011). Endothelial cells expressing DN-KASH alter cell–cell adhesion, barrier function, cell–matrix adhesion, and FA dynamics (Denis et al., 2021). In addition, nesprin-1 depletion in endothelial cells increases the number of FAs, cell traction force, and nuclear height (Chancellor et al., 2010). Conversely, nesprin-2G-knocked out fibroblasts, impaired in TAN line formation as well as the loss of cytoplasmic and perinuclear actin staining, exhibited decreased FA size, number, and expression of FA proteins, and reduced traction force (Woychek and Jones, 2019). Thus, the LINC complexes act as nuclear nodes that bidirectionally transmit signals between the cytoskeleton and the nucleus. However, the functional importance of SUN proteins in the maturation of FAs and the integrity of the actin cytoskeleton has never been directly examined.

In the present study, we investigated the effects of the depletion of SUN proteins on the actin cytoskeleton and FA maturation. We report that SUN1 is essential for proper actin organization, generation of intracellular traction force, and the maturation of FA. In addition, these data suggest that the elucidation of the mechanism by which the LINC complex transmits nuclear features such as epigenetic histone code and nuclear lamina architecture to the cytoskeleton will reveal its effects on diverse cellular functions.

## Materials and Methods

### Antibodies and Solutions

Rabbit anti-SUN1 polyclonal antibody (pAb) (HPA008346) was purchased from Sigma Aldrich (St. Louis, MO, United States) and used at 1:100 to 1:200 dilution for immunofluorescence microscopy. Rabbit anti-SUN2 pAb (06-1038) was obtained from Merck Millipore (Temecula, CA, United States) and used at a 1:200 dilution for immunofluorescence microscopy. Mouse anti-β-actin monoclonal antibody (mAb) clone AC-15 (A1978) and clone 6D1 were obtained from Sigma-Aldrich and Fujifilm Wako Pure Chemical Corporation (Osaka, Japan), respectively. Mouse anti-vinculin mAb (clones VIN11-5 and V4505) was purchased from Sigma Aldrich and used at a 1:300 dilution for immunofluorescence microscopy. Mouse anti-β-tubulin mAb (clones TUB2.1 and T4026) was purchased from Sigma-Aldrich. Mouse anti-integrin β1 mAb (TS2/16) was procured from eBIoscience, Inc. (San Diego, CA, United States) and Santa Cruz (Dallas, TX, United States), and used at 1:50 to 1:100 dilution for immunofluorescence microscopy. Mouse anti-active integrin β1 mAb (HUTS-4, MAB 2079Z) was purchased from Merck Millipore and used at a 1:50 dilution for immunofluorescence microscopy. Mouse anti-active integrin β1 mAb (12G10) was purchased from BioRad (Hercules, CA, United States) and used at 1:100 dilution for immunofluorescence microscopy. Rabbit anti-FAK pAb (#3285) and rabbit anti-Tyr397 phosphorylated focal adhesion kinase (FAK-pY397) mAb (D20B1) were purchased from Cell Signaling (Danvers, MA, United States) and used at 1:500 dilution for western blotting. Rabbit anti-FAK-pY397 pAb (sc-11765-R) was from Santa Cruz Biotechnology and used at 1:50 dilution for immunofluorescence microscopy. Mouse anti-paxillin mAb (D-9 sc365174 and B-2 sc365379) was obtained from Santa Cruz Biotechnology and used at 1:100 dilution for immunofluorescence microscopy. Mouse anti-Tyr118 phosphorylated paxillin mAb (A-5 sc365020) was obtained from Santa Cruz Biotechnology and used at 1:100 dilution for immunofluorescence microscopy. Mouse anti-zyxin mAb (sc-136128) was obtained from Santa Cruz Biotechnology and used at a 1:200 dilution for immunofluorescence microscopy. Anti-YAP (D8H1X) XP rabbit mAb was purchased from Cell Signaling and used at a 1:100 dilution for immunofluorescent microscopy. Rat anti-histone H3 mAb was a gift from Dr. H. Kimura (Tokyo Institute of Technology). Fluorescent secondary antibodies were obtained from Jackson ImmunoResearch Laboratories (Soham, United Kingdom). Rhodamine phalloidin was purchased from Cytoskeleton Inc. (Denver, CO, United States).

### Cell Culture and Transfection

HeLa cells were obtained from the Japanese Cancer Research Bioresources (JCRB) Cell Bank and grown in Dulbecco’s modified Eagle’s medium (low glucose, Fujifilm Wako Pure Chemical Corporation) supplemented with 10% fetal calf serum at 37°C in a 10% CO_2_ atmosphere. HeLa cells were used in this study unless otherwise stated. The human mammary epithelial cell line MCF10A (CRL-10317) was obtained from the American Type Culture Collection (ATCC) and cultured as previously described (Yokoyama et al., 2014). SUN1-knocked out HeLa cells have been described previously (Nishioka et al., 2016) and cultured as previously described. Transfection was performed using Gene Juice (Merck Millipore) and described previously (Satomi et al., 2020).

### siRNA-Mediated Knockdown

The sequences of siRNA pools against SUN1 (UNC84A) and SUN2 (UNC84B) have been described previously (Nishioka et al., 2016). The siRNAs were obtained from Nippon Gene (Tokyo, Japan). Cells were transfected with the indicated siRNAs or a non-targeting siRNA pool (siNC, Thermo Fisher Scientific, Waltham, MA) as a negative control using Lipofectamine RNAiMAX reagent (Invitrogen, CA, United States) as previously described (Hieda et al., 2021). Briefly, all siRNAs were used at a final concentration of 10 nM, and cells were fixed or harvested 48 h after transfection unless otherwise stated.

### Immunostaining and Quantification of Focal Adhesions

The cells were fixed with 4% paraformaldehyde and immunostaining was mostly performed as described previously using appropriate primary and secondary antibodies (Hieda et al., 2008) unless stated otherwise. For HUTS4 mAb staining, cells were fixed and stained without Triton X-100 permeabilization. Actin filaments were stained with 50 nM rhodamine–phalloidin for 30 min. For YAP staining, cells were cultured on type I collagen-coated cover glass (#4910-010, Iwaki, Japan). For Triton X-100 permeabilization before fixation, cells were washed twice with ice-cold transport buffer (TB, 20 mM HEPES, pH 7.3; 110 mM potassium acetate; 2 mM magnesium acetate; 5 mM sodium acetate; 0.5 mM EGTA; Adam et al., 1990) and subsequently incubated with TB containing 0.5% Triton X-100 for 5 min on ice, followed by fixation. Cells were viewed and captured with Olympus IX81 with Plan Apo 60×/NA1.4 or Olympus BX53 with a UPlanS Apo 40×/NA 0.95 objective lens using an Olympus DP-73 camera, Olympus U-HGLGPS light source, and Olympus U-FBNA filter (excitation 470–495, emission 510–550) and Olympus U-FGW (excitation 530–550, emission).

Quantification of the number, area, and fluorescent intensity was performed using ImageJ software (https://imagej.nih.gov/ij/). The number of FAs was quantified after thresholding and segmentation. The integrated density (i.e., the sum of all pixels in the ROI, region of interest) was measured as “RawIntDen”.

### Lysate Preparation and Western Blotting

The total cell extract was collected using 2× sample buffer and sonicated, or syringe sampled 15 times using a 1 ml syringe fitted with a 24 G needle to ensure lysate homogenization and genomic DNA shearing. Afterward, the protein concentration was analyzed using Ionic Detergent Compatibility Reagent (Thermo Fisher Scientific) and Pierce 660 nm Protein Assay Reagent (Thermo Fisher Scientific) according to the manufacturer’s instructions. The total cell lysate was analyzed by western blotting using the indicated antibodies.

### Adhesion Assay

Cell adhesion assays were performed as described previously (Hieda et al., 2015). Briefly, 96-well plates were coated with 100 μL of 5 μg/ml laminin (AGC Inc., Tokyo, Japan), 5 μg/ml vitronectin (Wako Pure Chemical, Osaka, Japan), 5 μg/ml fibronectin (AGC Inc.), 5 μg/ml collagen type IC (Nitta Gelatin, Osaka, Japan), or 3% bovine serum albumin (BSA) and blocked with 3% BSA. Next, cells were added to the plates. After 2 h of incubation at 37°C, plates were washed with phosphate-buffered saline (PBS) and cells were stained with crystal violet. The absorbance was measured using measurement filter 595 nm and reference filter 630 nm. Experiments were repeated at least four times.

### Integrin β1 Internalization and Recycling Assay

The internalization and recycling assays of integrin β1 were performed as described previously (Maekawa et al., 2017). Briefly, integrin β1 on the cell surface was labeled with Alexa 488-conjugated TS2/16 antibody in the growth medium containing 30 mM HEPES (pH 7.6) on ice for 1 h. Next, the cells were washed with ice-cold PBS and the medium was replaced with a fresh growth medium containing 30 mM HEPES (pH 7.6). Cells were incubated at 37°C for the indicated time (mentioned in the figure caption) to allow the internalization of fluorescent integrin β1. After internalization, the remaining fluorescence on the cell surface was quenched with an anti-Alexa 488 antibody. For the internalization assay, cells were fixed, and the signals inside the cells were imaged. To monitor the recycling of integrin β1, cells were re-incubated at 37°C for the indicated time points. After re-incubation, the surface fluorescence signal of integrin β1 was quenched again. Cells were subsequently fixed, and the signals inside the cells were imaged. For both internalization and recycling assays, images were quantified using the ImageJ software.

### Evaluation of Traction Force

Traction force was visualized using wrinkle formation assay as described previously (Ichikawa et al., 2017; Kang et al., 2020). Briefly, silicone substrates CY 52-276 (Dow Corning Toray, Tokyo, Japan) were mixed at a weight ratio of 1.2:1 and spread on a coverslip to have a final elastic modulus of 5.4 kPa. To measure the elastic modules in a separate experiment with the Hertz contact model (Ichikawa et al., 2017), stainless beads were placed onto the substrate where the surface was coated in advance with fluorescent microbeads through silane coupling. For this process, the silicone surface was treated with 2% (3-aminopropyl) trimethoxysilane (Sigma Aldrich) in 90% EtOH for 30 min and then with 20% glutaraldehyde (Wako) for 5 min. By taking 3-dimensional images of the fluorescently labeled substrate using a confocal laser scanning microscope (FV-1000; Olympus), the indentation depth associated with the elastic modulus was measured. The coverslip was exposed to 4 mA oxygen plasma for hydrophilization for 1 min using a plasma generator (SEDE-GE, Meiwafosis, Tokyo, Japan), put on a 6-well culture plate, and coated with 10 µg/ml fibronectin (Sigma-Aldrich), on which HeLa cells (parental wild-type or SUN1-depleted) were cultured at 37°C in a 5% CO_2_ stage incubator mounted on an inverted microscope (IX71; Olympus, Tokyo, Japan). 48 h after incubation, cells with wrinkle formation were imaged with phase-contrast microscopy using a ×10 semi-apochromat objective lens (NA 0.3). The acquired images were analyzed to automatically detect cellular traction force-generated wrinkles using a custom-made program written in Fiji software. Briefly, images were processed with a two-dimensional fast Fourier transformation and then with a band-pass filter to extract the wrinkles, which were skeletonized into line segments and integrated to finally obtain their total length (the number of pixels) per cell as traction index.

## Results

### Depletion of SUN1 Affects Actin Organization

**FIGURE 1:**
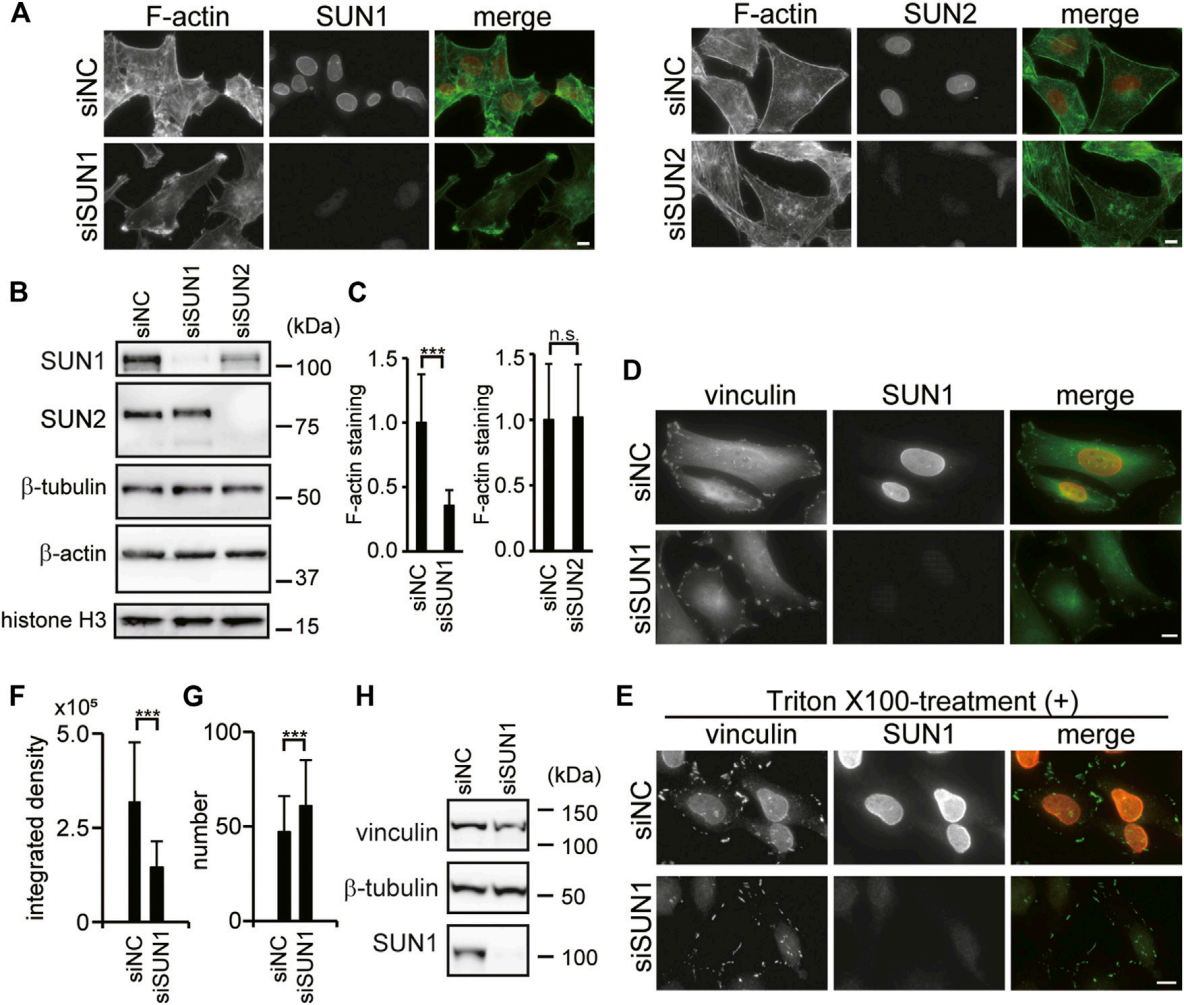
SUN1 depletion affects actin filament and suppresses vinculin incorporation into FAs. **(A)** HeLa cells were transfected with siRNA against SUN1 (siSUN1), SUN2 (siSUN2), or negative control siRNA (siNC) and stained with rhodamine–phalloidin and anti-SUN1 or anti-SUN2 pAb. Scale bar, 10 μm. **(B)** Cells were transfected with indicated siRNAs. The expression of SUN1, SUN2, β-tubulin, histone H3, and β-actin proteins in the total cell lysate was analyzed by western blotting. **(C)** Actin staining intensity in the cytoplasm was quantified. The values represent the mean of relative intensity to the siNC-transfected cells ±standard deviation (SD). ***p < 0.005 compared with the siNC-transfected cells. **(D)** Cells were transfected with siSUN1 or siNC, and next fixed and stained with anti-vinculin mAb and anti-SUN1 pAb. Scale bar, 10 μm. **(E)** Cells were transfected with siSUN1 or siNC and treated with 0.5% Triton X-100 for 5 min on ice. Cells were subsequently fixed and stained with anti-vinculin mAb and anti-SUN1 pAb. Scale bar, 10 μm. **(F**,**G)** Triton X-100 resistant vinculin signal in siNC and siSUN1 transfected cells were quantified using the ImageJ software. The values represent the mean of the integrated density of vinculin **(F)** and the number of vinculin dots per cell **(G)** ± standard deviation (SD). *N* > 100. ***p < 0.005 compared with siNC-transfected cells. **(H)** Cells were transfected with siSUN1 or siNC. The expression of vinculin, β-tubulin and SUN1 proteins was analyzed by western blotting.

SUN1 is required for directional cell migration (Nishioka et al., 2016; Imaizumi et al., 2018); however, the underlying mechanism remains unknown. Both SUN1 and SUN2 proteins promiscuously interact with nesprin-2 and form the LINC complex (Stewart-Hutchinson et al., 2008; Ostlund et al., 2009; Haque et al., 2010; Sosa et al., 2012). Because nesprin-2G physically connects with the actin cytoskeleton (Zhen et al., 2002; Luxton et al., 2010), and its depletion reduces the ability to generate traction force (Woychek and Jones, 2019), we examined the involvement of SUN1 and SUN2 in the organization of the actin cytoskeleton using siRNAs. Depletion of SUN1 and SUN2 proteins was confirmed by immunofluorescence microscopy and western blotting (Figures 1A,B). Quantitative analysis showed that more than 90% of SUN1 and SUN2 expressions were depleted in each siRNA-targeted knockdown cell. Interestingly, cytoplasmic actin staining in the SUN1- but not SUN2-depleted cells decreased compared with the control cells (Figures 1A,C and Supplementary Figure S1A). Reduced staining intensity in the SUN1-depleted cells was rescued by the expression of mouse SUN1, which is siSUN1 resistant (Supplementary Figure S1B). In addition, some of the SUN1-depleted cells potentially have increased actin ruffling at their periphery (Figure 1A and Supplementary Figure S1A arrows), while obvious stress fibers and sub-nuclear actin structures were observed in the SUN2-depleted cells but not in the control cells (Figure 1A and Supplementary Figure S1C). However, the relative expression of β-actin protein remained the same in the control cells and the SUN1- or SUN2-depleted cells (Figure 1B and Supplementary Figure S1D). This is consistent with previous reports (Thakar et al., 2017). Thakar et al. reported that the mRNA levels of β-actin remained unaffected by the depletion of SUN1 in HeLa cells (Thakar et al., 2017). These results suggest that SUN1 depletion affects the organization of filamentous actin. Thus, we focused on the function of SUN1 in subsequent experiments.

### SUN1-Depletion Suppresses Vinculin Incorporation Into Focal Adhesions

Directional cell migration requires continuous turnover of FAs along the direction of cell movement (Lock and Strömblad, 2008). Thus, the effects of SUN1 depletion on FAs were examined by observing the localization of vinculin, a cytoskeletal adaptor protein in FAs (Carisey and Ballestrem 2011). Depletion of SUN1 was confirmed by immunofluorescence microscopy (Figure 1D). Accumulated vinculin signal was observed in the periphery of both control and SUN1-depleted cells (Figure 1D) indicating that vinculin is recruited to the plasma membrane in both control and SUN1-depleted cells. Of note, we did not observe an elevated level of plasma membrane-localized vinculin in the SUN1-depleted cells, while Thakar et al. (2017) showed that SUN1-depleted HeLa cells have increased vinculin staining at the plasma membrane as well as an increased level of GTP-bound RhoA. This disparity could be caused by activation by fibronectin on the coverslips they used. We did not observe an elevated level of GTP-RhoA in the SUN1-depleted cells (Supplementary Figure S1E).

Because the SUN1-depleted cells show many long cellular processes and the accumulation of actin at the tips of these processes that are not present in the control cells (Figure 1A, Supplementary Figure S1A, and Supplementary Figure S1C), we analyzed the effects of SUN1 depletion on FA turn over using a microtubule-induced FA disassembly assay (Ezratty et al., 2009). The result showed that SUN1-depleted cells retain the ability to disassemble their FAs (Supplementary Figure S2A). Vinculin has two distinct conformations, namely “open form” and “closed form” (Bays and DeMali, 2017). A significant amount of vinculin is a closed form at steady-state and can be washed out by Triton X-100 treatment, whereas a certain amount of vinculin is Triton X-100 insoluble because it is incorporated into FAs and binds to actin filaments (Lee and Otto, 1997; Sawada and Sheetz, 2002; Yamashita et al., 2014). To explore the incorporation of vinculin into FAs, cells were treated with Triton X-100 before fixation and stained with an anti-vinculin antibody. Triton X-100 treatment caused the dispersion of vinculin signals into small punctate patterns in SUN1-depleted cells, whereas condensed vinculin signals in siNC-transfected cells were resistant to Triton X-100 treatment (Figure 1E). The dispersion of the vinculin signal in the SUN1-depleted cells was rescued by the expression of siSUN1-resistant mouse SUN1 (Supplementary Figure S2B). Quantified data indicated that the integrated density of Triton X-100-resistant vinculin was decreased in SUN1-depleted cells compared with that in the control cells (Figure 1F), whereas the number of vinculin-positive dots was increased in SUN1-depleted cells (Figure 1G). The protein expression of vinculin in the SUN1-depleted cells remained unaltered (Figure 1H and Supplementary Figure S2C). Therefore, these results indicate that SUN1 depletion does not interfere with the recruitment of vinculin to the plasma membrane; however, it suppresses the conformational change in vinculin to a Triton X-100-resistant form (i.e., incorporation into FAs), which requires cytoskeletal forces (Omachi et al., 2017). Of note, SUN1 was resistant to Triton X-100 treatment (Figure 1E, upper panel), probably because SUN1 associates with nesprins, lamins, and/or chromatin.

### SUN1 Depletion Reduces Active Integrin β1 Levels

**FIGURE 2:**
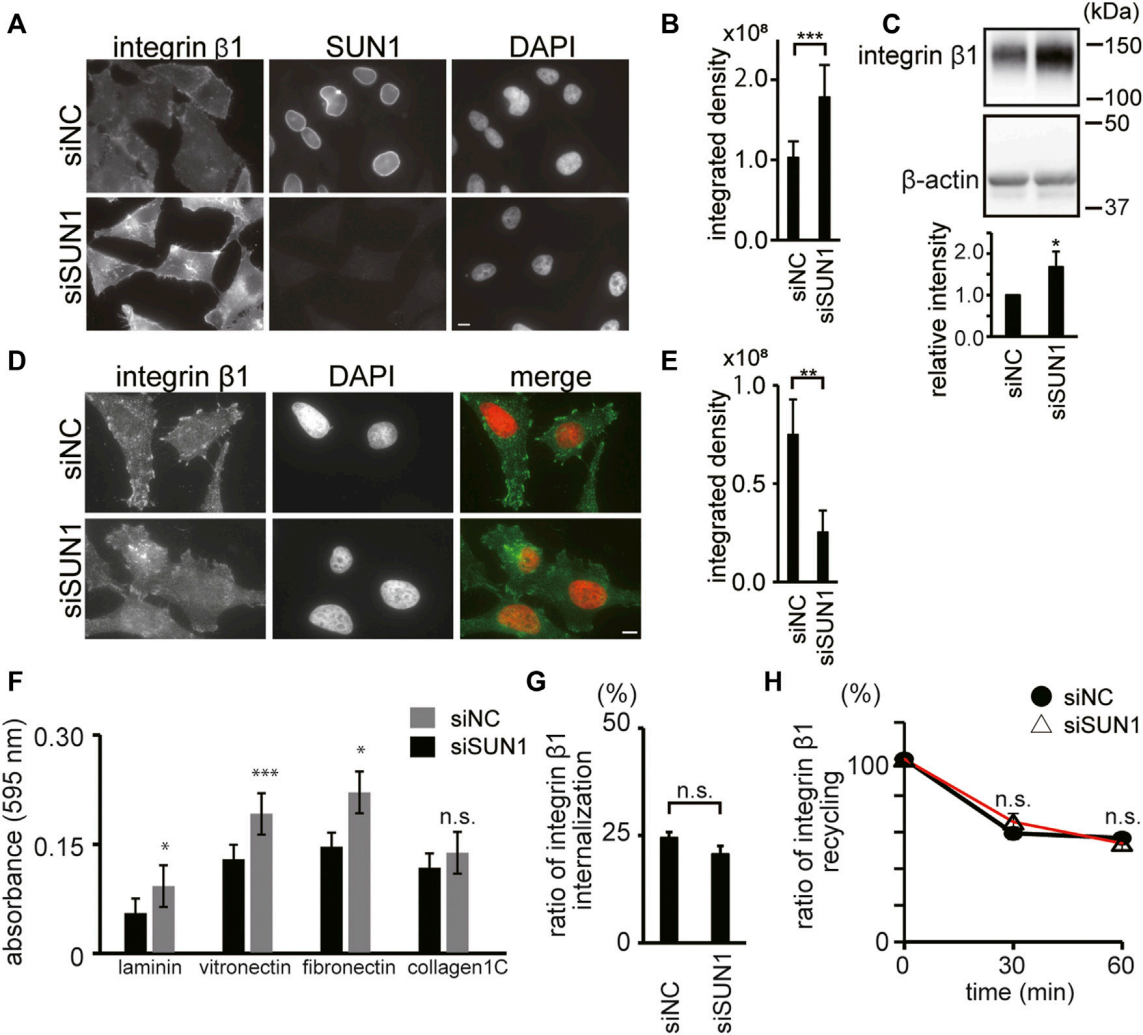
SUN1 depletion reduces active integrin β1. **(A)** Cells were transfected with siSUN1 or siNC. Next, the cells were fixed and stained with anti-integrin β1 mAb (TS2/16), which recognizes both its active and inactive forms and anti-SUN1 pAb. Scale bar, 10 μm. **(B)** The integrated density of the total integrin β1 staining was quantified using the ImageJ software. The values represent the mean ± standard deviation (SD). *N* > 100. ***p < 0.005 compared with siNC-transfected cells. **(C)** Cells were transfected with siSUN1 or siNC. The cell lysate was analyzed by western blotting using anti-integrin β1 mAb (TS2/16) and anti-β-actin mAbs. The values represent the mean of the relative intensity of integrin β-1 expression to β-tubulin in the western blotting ±standard deviation (SD). **(D)** Cells were transfected with siSUN1 or siNC. Next, the cells were fixed and stained with anti-active integrin β1 mAb (HUTS4). Note that the epitope of HUTS4 is localized in the extracellular domain of integrin β1. The Triton X-100 permeabilization step was eliminated from the staining process in the case of HUTS4 mAb staining because it greatly increases the non-specific signal in the cytoplasm. Scale bar, 10 μm. **(E)** The integrated density of the active integrin β1 staining was quantified using the ImageJ software. The values represent the mean ± standard deviation (SD). *N* > 100. **p < 0.01 compared with siNC-transfected cells. **(F)** Cells were transfected with siSUN1 or siNC. Next, the cell–extracellular matrix (ECM) adhesion activity was measured using cell culture plates coated with fibronectin, vitronectin, laminin, or collagen type IC. Experiments were repeated four times and a representative result is shown. ***p < 0.005, *p < 0.05 compared with siNC-transfected cells. n.s., not significant. **(G)** The rate of internalization of integrin β1 was analyzed. Cells were transfected with siSUN1 or siNC. After 48 h of incubation, cell surface integrin β1 was labeled with Alexa 488-conjugated TS2/16 mAb and chased for 10 min. Next, the remaining cell surface fluorescent was quenched. The ratio of fluorescence intensity inside the cell to that on the cell surface before chasing is shown. (*n* = 30). The values represent the mean ± standard error of the mean (SEM). n.s., not significant. Representative images are shown in Supplementary Figure S4A. **(H)** Recycling of integrin β1 was analyzed in the SUN1-depleted cells. Cells were treated with siSUN1 or siNC. After 48 h of incubation, cell surface integrin β1 was labeled with Alexa 488 conjugated to TS2/16 mAb and chased for 60 min to allow endocytosis. Next, the remaining fluorescent at the cell surface was quenched (time 0) and cells were incubated to allow trafficking from endosomes to the plasma membrane. After the indicated incubation periond, cell surface fluorescence was again quenched. Representative images are shown in Supplementary Figure S4B. The fluorescence intensity inside the cells was measured and the intensity of Alexa 488-TS2/16 is shown as a percentage of that at 0 min (*n* = 30). Data represent the mean ± standard error of the mean (SEM). n.s., not significant.

To explore the maturation of FAs in SUN1-depleted cells, we next focused on integrin β1, which is a ubiquitously expressed FA molecule that drives the establishment of nascent adhesion sites (Schiller et al., 2013). To examine the availability of integrin β1 at the plasma membrane, we stained integrin β1 in SUN1-depleted cells using an anti-integrin β1 mAb, TS2/16 (Sanchez-Madrid et al., 1982), which recognizes both active and inactive forms of integrin β1. The intensity of total integrin β1 (i.e., the sum of active and inactive forms) at the plasma membrane in SUN1-depleted cells was obviously increased (Figures 2A,B). In addition, increased intensity of cell surface integrin β1 was observed following SUN1 depletion in both the non-cancerous breast epithelial cell line MCF10A (Supplementary Figure S3A) and unfixed cells (Supplementary Figure S4A; 0 min). Western blotting showed more than a 1.5 times increase in integrin β1 protein expression in the SUN1-depleted cells (Figure 2C).

The activation of integrins promotes the recruitment of several adaptor proteins to form submicrometer clusters and trigger FA maturation (Bouvard et al., 2013). Because the balance between active and inactive integrins is dynamically regulated (Khan and Goult, 2019), we analyzed the active form of integrin β1 using an anti-integrin β1 mAb, HUTS4 (Luque et al., 1996), which recognizes only the active form of integrin β1. Intriguingly, the intensity of cell surface-active integrin β1 was decreased in SUN1-depleted cells (Figure 2D). HUT4-positive large structures recognized in siNC-transfected cells disappeared in siSUN1-transfected cells, and weak filamentous structures were observed (Figure 2D). Quantification of staining intensity showed a significant reduction in active integrin β1 intensity in SUN1-depleted cells (Figure 2E). In addition, a ligand-binding form of integrin β1 in the control cells was colocalized with vinculin and these signals were Triton X-100 resistant (Supplementary Figure S3B). The decreased active integrin β1 in the SUN1-depleted cells was recovered by the expression of siSUN1-resistant mouse SUN1 (Supplementary Figure S3C).

Cell adhesion to the ECM activates integrins (Shattil et al., 2010). Because SUN1-depleted cells showed diminished cell surface-active integrin β1 (Figures 2D,E), the depletion of SUN1 could attenuate the adhesion activity of the cells. However, in contrast to our expectations, SUN1-depleted cells showed slightly but reproducibly increased adhesion activity (Figure 2F). This is in agreement with the upregulation of integrin β1 expression at the plasma membrane (Figures 2A–C) and indicates that impaired adhesion is not responsible for the reduction of activated integrin β1 at the cell surface.

We next studied the effect of SUN1-depletion on the intracellular trafficking of integrin β1. Integrins continuously cycle between the plasma membrane and internal compartments with low lysosomal degradation rates (Lobert et al., 2010; Nader et al., 2016). The amount of active integrin β1 at the plasma membrane is regulated by the rate of endocytosis from the plasma membrane and recycling from endosomes to the plasma membrane (De Franceschi et al., 2015). Thus, facilitated internalization of integrin β1 from the plasma membrane or its aberrant recycling to the cell surface could decrease the cell surface-active integrin β1. However, depletion of SUN1 affected neither the internalization efficiency of integrin β1 (Figure 2G) nor the recycling efficiency to the cell surface (Figure 2H). Therefore, these data indicate that SUN1 is not involved in the trafficking of integrin β1. Based on these findings, we assume that the reduction of active integrin β1 in SUN1-depleted cells could be related to the impaired actin cytoskeleton (Figure 1A) because physical forces exerted by actin fibers are transmitted to the cytoplasmic domain of integrins, thus activating it.

### Depletion of SUN1 Abrogates the Maturation of Focal Adhesions at the Cytoskeletal Force-Dependent Step

**FIGURE 3:**
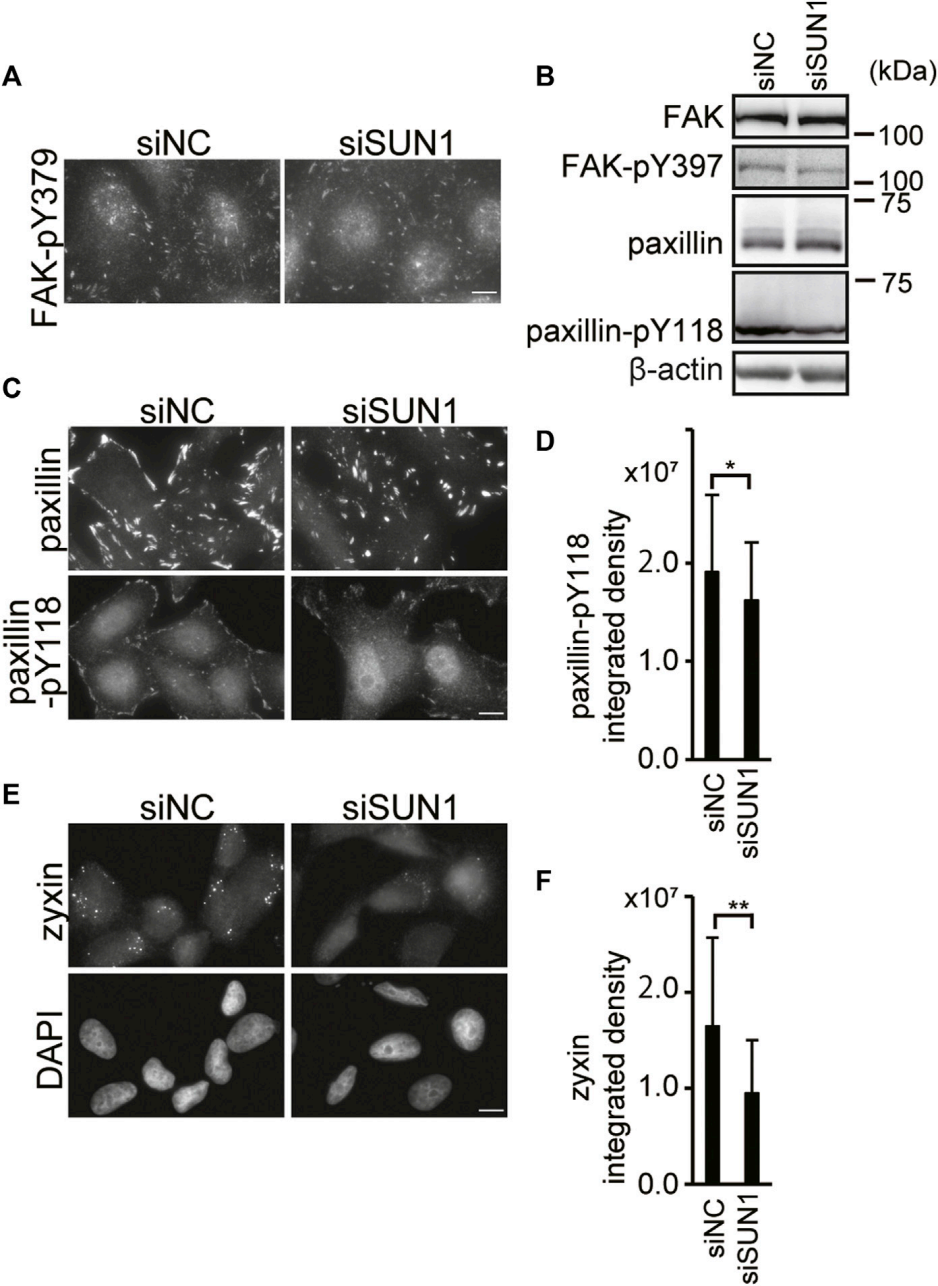
SUN1 depletion suppresses FA maturation. **(A)** Cells were transfected with siSUN1 or siNC. Next, the cells were fixed and stained with anti-Tyr379 phosphorylated FAK mAb. Scale bar, 10 μm. **(B)** Cells were transfected with siSUN1 or siNC. Afterward, the cell lysate was analyzed by western blotting using anti-FAK, anti-FAK-pY397, anti-paxillin, anti-paxillin-pY118, and anti-β-actin mAbs. **(C)** Cells were transfected with siSUN1 or siNC and stained with anti-paxillin or anti-paxillin-pY118 mAb. Scale bar, 10 μm. **(D)** The integrated density of the Tyr118 phosphorylated paxillin staining was quantified using the ImageJ software. The values represent the mean ± standard deviation (SD). *p < 0.05 compared with siNC-transfected cells. **(E)** Cells were transfected with siSUN1 or siNC. Next, the cells were fixed and stained with anti-zyxin mAb. Bar, 10 μm. **(F)** The integrated density of the zyxin staining was quantified. The values represent the mean ± standard deviation (SD). **p < 0.01 compared with siNC-transfected cells.

Depletion of SUN1 suppressed the incorporation of vinculin into FAs and the activation of integrin β1. The maturation of FA involves a stereotypical sequence of protein recruitment (Kuo et al., 2011). The initial stages of adhesion assembly occur in the actin-rich region at the cell periphery. Then, clustering of integrins occurs to form nascent adhesion, which is myosin-II independent. Also, additional FA proteins such as vinculin, phosphorylated paxillin, and FAK are recruited in a process termed maturation (Zaidel-Bar et al., 2003; Oakes and Gardel, 2014). To examine which step in FA maturation is disrupted in SUN1-depleted cells, we visualized FAs using antibodies against several FA-resident proteins, such as Tyr397 phosphorylated FAK (FAK-pY397), paxillin, Tyr118 phosphorylated paxillin (paxillin-pY118), and zyxin. FAK is a key tyrosine kinase involved in integrin signaling (Schlaepfer et al., 1999). The binding of integrin to the ECM and its clustering trigger the autophosphorylation of FAK at Tyr397 (Schlaepfer et al., 1999), which is critical for the maturation of FA; however, it is independent of mechanical tension (Horton et al., 2016). Depletion of SUN1 did not influence the FAK-pY397 staining intensity although the staining pattern was slightly altered (Figure 3A). Moreover, western blotting showed no significant differences in the expression of FAK protein and FAK Tyr397 phosphorylation between SUN1-depleted and control cells (Figure 3B). In addition, SUN1 depletion did not affect the protein expression or staining intensity of paxillin (Figures 3B,C), which is a scaffold protein in FAs and recruited to newly formed adhesions in a tension-independent manner. In contrast, the level of paxillin-pY118, which depends on intracellular traction force (Pasapera et al., 2010), moderately decreased in the SUN1-depleted cells (Figures 3B–D). Moreover, zyxin staining largely disappeared in SUN1-depleted cells, whereas zyxin staining patterns in control cells showed a relatively large dotted distribution (Figures 3E,F). The decreased zyxin signal in the SUN1-depleted cells was rescued by the expression of siSUN1-resistant mouse SUN1 (Supplementary Figure S3C). Because intracellular traction forces drive FA growth and recruitment of FA proteins such as zyxin (Zaidel-Bar et al., 2003), these results suggest a reduction in intracellular forces in SUN1-depleted cells.

### SUN1 is Involved in the Generation of Intracellular Forces

**FIGURE 4:**
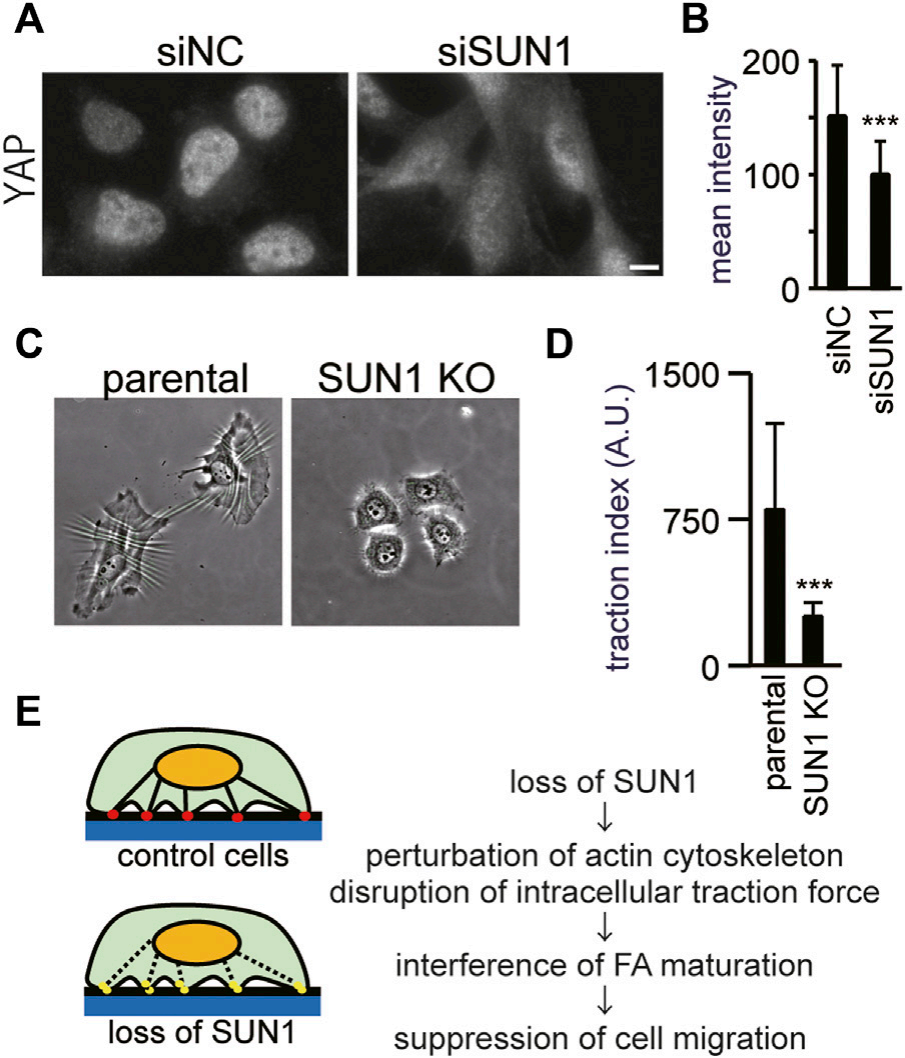
SUN1 is involved in the generation of intracellular forces. **(A)** Cells were transfected with siSUN1 or siNC and next stained with anti-YAP mAb. Scale bar, 10 μm. **(B)** The average staining intensity of YAP was measured (*n* > 60). The values represent the mean intensity ±standard deviation (SD). ***p < 0.001 compared with siNC-transfected cells. **(C)** Wrinkle formation assay was performed using SUN1 knocked out HeLa cells (Nishioka et al., 2016). In this assay, the contractile forces were visualized by wrinkle formation. **(D)** Quantification of the traction force-driven wrinkles (the number of pixels associated with the wrinkles) per individual cell was performed. Every test was conducted as a paired experiment to determine the relative contribution of SUN1 expression to the generation of cellular contractile forces. Data represent the mean ± standard deviation (SD). ***p < 0.001 compared with control (parental wild-type) cells. **(E)** Working model for the intracellular forces and FA maturation by SUN1. The control cells produce contractile force, mature focal adhesion, and migrate normally (upper panel). In contrast, SUN1 depletion affects actin organization and the SUN1-depleted cells do not produce contractile force, suppress FA maturation, and do not migrate (lower panel).

Depletion of SUN1 did not prevent the recruitment of inactive integrin β1 and vinculin at the plasma membrane or inhibit the autophosphorylation of FAK at Tyr397. In contrast, the loss of SUN1 reduced the number of FAs that contain vinculin and active integrin β1. These results suggest that the depletion of SUN1 suppresses the force-dependent step of FA maturation. Thus, to investigate the intracellular forces, we first visualized the nuclear localization of a transcriptional co-regulator, Yes-associated protein (YAP). Because YAP enters the nucleus in an intracellular force-dependent manner in several cells including HeLa (Dupont et al., 2011; Finch-Edmondson and Sudol, 2016), it can be used as an indicator of intracellular forces. As reported previously (Panciera et al., 2017), YAP proteins were mostly present in the nucleus in control cells cultured on a glass coverslip (Figure 4A, left panel) because of the rigidity of glass as a substrate. In contrast, the depletion of SUN1 decreased the YAP signal in the nucleus and increased it in the cytoplasm (Figure 4A, right panel). The average staining intensity of YAP in the SUN1-depleted nucleus was decreased as compared with that in siNC-transfected cells (Figure 4B), supporting a reduction in cytoskeletal forces.

Next, we directly assessed the effects of depletion of SUN1 on the generation of traction force, which can be assayed by assessing the ability of cells to induce the formation of wrinkles on deformable silicone substrates (Ichikawa et al., 2017; Kang et al., 2020), because the contractile forces within a cell correlate with the length of wrinkles (Burton and Taylor, 1997; Fukuda et al., 2017). The control cells exhibited extensive wrinkles on the substrate, whereas the loss of SUN1 suppressed the formation of wrinkles, indicating the significantly decreased contractile forces in SUN1-depleted cells (Figures 4C,D). Because phase contrast images show differences in cell spreading between the SUN1-depleted and control cells (Figure 4C), the areas of cell spreading were quantified. The SUN1-depleted cells on the silicone substrates showed less spreading than the control cells; the SUN1-depleted cells on the glass coverslips also had less spreading, but the effect was moderate (Supplementary Figure S5), suggesting a possible potential issue with mechanosensing.

## Discussion

In the present study, we demonstrated that the loss of SUN1 increases actin ruffling at their periphery and decreases cytoplasmic F-actin. In addition, loss of SUN1 weakened the intracellular forces under normal growth conditions. The maturation of FAs in SUN1-depleted cells was impaired at the force-dependent step, such as activation of integrin β1 and incorporation of vinculin and zyxin. In contrast, the loss of SUN1 did not affect the levels of FAK phosphorylation at Tyr397 or the recruitment of a Triton X-100-soluble form of vinculin to the plasma membrane, both of which occur at the early stage of FA formation in a force-independent manner. Based on these results, we propose a model of how the inner nuclear membrane protein, SUN1, participates in the maturation of FAs and cell migration under normal growth conditions (Figure 4E). Cells produce a contraction force by the actin cytoskeleton that connects FAs and the LINC complex (Figure 4E, upper panel). The depletion of SUN1 perturbs the proper actin organization, thereby abrogating the generation of contraction force (Figure 4E, lower panel). This effect on the actin cytoskeleton suppressed the maturation of FAs and cell migration. Therefore, the LINC complexes are critical not only for transmitting mechanical information from the cytoplasm to the nucleus but also for force-dependent cytoplasmic functions, such as the maturation of FAs. In addition, the findings of the present study have two important implications for our understanding of how the LINC complex functions in diverse physiological and pathological processes.

First, SUN1 is an essential factor in actin organization and intracellular traction force. This is consistent with previous data showing that disruption of nesprin-1 or nesprin-2 altered the actin cytoskeleton and reduced the ability to generate traction force (Chancellor et al., 2010; Lombardi et al., 2011; Woychek and Jones, 2019). Woychek and Jones (2019) showed that nesprin-2G knockout reduced the ability of fibroblasts to exert traction force on their substrates relative to control cells. In addition to these data, we found that loss of SUN1 perturbed actin organization in the HeLa cells and reduced their ability to generate traction forces on their substrates, suggesting that SUN1/nesprin-2G-containing LINC complexes are key regulators of actin cytoskeletal organization. Moreover, with regard to the LINC complex-associated cytoskeleton, it has been shown that SUN1-containing LINC complexes preferentially interact with microtubules, and SUN2-containing LINC complexes preferentially interact with actin networks during the homeostatic positioning of nuclei in fibroblasts (Zhu et al., 2017). In addition, transmembrane actin-associated nuclear (TAN) lines are identified by the accumulation of nesprin-2G and SUN2 along the perinuclear actin cables on the dorsal nuclear surface of fibroblasts (Luxton et al., 2010). In contrast to these preferences of SUN1- and SUN2-containing LINC complexes to actin and microtubules, our data demonstrated the critical function of SUN1 in actin organization, suggesting that SUN1-containing LINC complexes function differently in association with actin or microtubules in different cellular contexts. The present study highlights the differential functions of SUN1 and SUN2 proteins although both proteins promiscuously interact with nesprins to form the LINC complex (Padmakumar et al., 2005; Crisp et al., 2006; Ketema et al., 2007; Stewart-Hutchinson et al., 2008; Ostlund et al., 2009).

Second, the results of this study suggest a contribution of the loss of the LINC complex to cancer progression. We have previously reported the global loss of the LINC complex components, including SUN1 and nesprin-2, in human breast cancer tissues (Matsumoto et al., 2015). Analysis using The Cancer Genome Atlas (TCGA) and the Genotype-Tissue Expression (GTEx) datasets showed downregulated expression of SUN1 and SUN2 across tumor types (Sharma et al., 2021). In addition, a study of 3,000 cancer genomes across nine cancer types identified mutations in the *SYNE-1* gene encoding nesprin-1 as “drivers” in the development of cancer (Cheng et al., 2015). However, the mechanism of how the loss of the LINC complex components or mutated LINC complex affects cancer progression has remained elusive. In this study, the expression of integrin β1 was significantly enhanced in SUN1-depleted MCF10A and HeLa cells. Altered expression of integrin is frequently observed in tumor cells and is associated with poor clinical outcomes and cancer progression (Hamidi and Ivaska, 2018). For instance, integrins function in oncogenic growth factor receptor signaling, facilitate anchorage-independent survival of circulating tumor cells, and determine the colonization of metastatic sites. Upregulated integrin β1 in leading cells plays a role in collective cell migration—a common feature of metastatic cancer cells (Kato et al., 2014). Over-expression of integrins associates with increased formation of metastases in several tumors (Yoshimasu et al., 2004; Tsuji et al., 2002; Sordat et al., 2002). In addition, the upregulated integrin β1 could be involved in LINC-independent cell migration (Fracchia et al., 2020). Thus, these effects of increased expression of integrin β1 in the SUN1-depleted cells may contribute to cancer progression. The LINC complex has been shown to transfer mechanical stresses from the cytoskeleton to the genome to regulate gene expression (Chancellor et al., 2010; Simon and Wilson, 2011; Rashmi et al., 2012; Alam et al., 2016), possibly through chromatin remodeling (Iyer et al., 2012; Booth et al., 2015; Toh et al., 2015), dissociation of protein complexes inside the nucleus (Poh et al., 2012), and motion of intranuclear organelles (Zhang et al., 2016) although the underlying molecular mechanism of transcriptional regulation by the LINC complex is largely unknown. The increased expression of integrin β1 protein in SUN1-depleted cells is caused by transcriptional regulation because it is widely accepted that lysosomal degradation of integrin β1 is prevented under normal growth conditions (Böttcher et al., 2012; Steinberg et al., 2012). Integrin β1 is encoded by the *ITGB1* gene and its expression is regulated by myocardin-related transcription factor (MRTF)-A (also called Mkl1, Bsac, or Mal) and MRTF-B (also called Mkl2), which are transcriptional cofactors that work with serum response factor (SRF) (Miano et al., 2007). Studies conducted using a mouse model have reported that the loss of SUN1 increases the expression of SUN2 (Chen et al., 2012; Wang et al., 2015), which is also regulated in a SRF-dependent manner (May and Carroll, 2018). Thus, depletion of SUN1 may activate the expression of *ITGB1* either *via* MRTF-A or MRTF-B. Altogether, the present study sheds light on the contribution of SUN proteins to transcriptional regulation.

## Data Availability

The original contributions presented in the study are included in the article/Supplementary Material, further inquiries can be directed to the corresponding author.

## References

[B1] Adam S. A. Marr R. S. Gerace L. (1990). Nuclear Protein Import in Permeabilized Mammalian Cells Requires Soluble Cytoplasmic Factors. J. Cell Biol. 111, 807–816. 10.1083/jcb.111.3.807 2391365 PMC2116268

[B2] Alam S. G. Lovett D. Kim D. I. Roux K. J. Dickinson R. B. Lele T. P. (2015). The Nucleus Is an Intracellular Propagator of Tensile Forces in NIH 3T3 Fibroblasts. J. Cell Sci. 128, 1901–1911. 10.1242/jcs.161703 25908852 PMC4457156

[B3] Alam S. G. Zhang Q. Prasad N. Li Y. Chamala S. Kuchibhotla R. (2016). The Mammalian LINC Complex Regulates Genome Transcriptional Responses to Substrate Rigidity. Sci. Rep. 6, 38063. 10.1038/srep38063 27905489 PMC5131312

[B4] Arsenovic P. T. Ramachandran I. Bathula K. Zhu R. Narang J. D. Noll N. A. (2016). Nesprin-2G, a Component of the Nuclear LINC Complex, Is Subject to Myosin-dependent Tension. Biophysical J. 110, 34–43. 10.1016/j.bpj.2015.11.014 PMC4805861 26745407

[B5] Bays J. L. DeMali K. A. (2017). Vinculin in Cell-Cell and Cell-Matrix Adhesions. Cell. Mol. Life Sci. 74, 2999–3009. 10.1007/s00018-017-2511-3 28401269 PMC5501900

[B6] Birks S. Uzer G. (2021). At the Nuclear Envelope of Bone Mechanobiology. Bone 151, 116023. 10.1016/j.bone.2021.116023 34051417 PMC8600447

[B7] Booth E. A. Spagnol S. T. Alcoser T. A. Dahl K. N. (2015). Nuclear Stiffening and Chromatin Softening with Progerin Expression Leads to an Attenuated Nuclear Response to Force. Soft Matter 11, 6412–6418. 10.1039/c5sm00521c 26171741

[B8] Böttcher R. T. Stremmel C. Meves A. Meyer H. Widmaier M. Tseng H.-Y. (2012). Sorting Nexin 17 Prevents Lysosomal Degradation of β1 Integrins by Binding to the β1-integrin Tail. Nat. Cell Biol. 14, 584–592. 10.1038/ncb2501 22561348

[B9] Bouvard D. Pouwels J. De Franceschi N. Ivaska J. (2013). Integrin Inactivators: Balancing Cellular Functions *In Vitro* and *In Vivo* . Nat. Rev. Mol. Cell Biol. 14, 430–442. 10.1038/nrm3599 23719537

[B10] Burton K. Taylor D. L. (1997). Traction Forces of Cytokinesis Measured with Optically Modified Elastic Substrata. Nature 385, 450–454. 10.1038/385450a0 9009194

[B11] Carisey A. Ballestrem C. (2011). Vinculin, an Adapter Protein in Control of Cell Adhesion Signalling. Eur. J. Cell Biol. 90, 157–163. 10.1016/j.ejcb.2010.06.007 20655620 PMC3526775

[B12] Chancellor T. J. Lee J. Thodeti C. K. Lele T. (2010). Actomyosin Tension Exerted on the Nucleus through Nesprin-1 Connections Influences Endothelial Cell Adhesion, Migration, and Cyclic Strain-Induced Reorientation. Biophysical J. 99, 115–123. 10.1016/j.bpj.2010.04.011 PMC2895377 20655839

[B13] Chen C.-Y. Chi Y.-H. Mutalif R. A. Starost M. F. Myers T. G. Anderson S. A. (2012). Accumulation of the Inner Nuclear Envelope Protein Sun1 Is Pathogenic in Progeric and Dystrophic Laminopathies. Cell 149, 565–577. 10.1016/j.cell.2012.01.059 22541428 PMC3340584

[B14] Cheng F. Liu C. Lin C.-C. Zhao J. Jia P. Li W.-H. (2015). A Gene Gravity Model for the Evolution of Cancer Genomes: A Study of 3,000 Cancer Genomes across 9 Cancer Types. PLOS Comput. Biol. 11, e1004497. 10.1371/journal.pcbi.1004497 26352260 PMC4564226

[B15] Cho S. Irianto J. Discher D. E. (2017). Mechanosensing by the Nucleus: from Pathways to Scaling Relationships. J. Cell Biol. 216, 305–315. 10.1083/jcb.201610042 28043971 PMC5294790

[B16] Crisp M. Liu Q. Roux K. Rattner J. B. Shanahan C. Burke B. (2006). Coupling of the Nucleus and Cytoplasm: Role of the LINC Complex. J. Cell Biol. 172, 41–53. 10.1083/jcb.200509124 16380439 PMC2063530

[B17] De Franceschi N. Hamidi H. Alanko J. Sahgal P. Ivaska J. (2015). Integrin Traffic - the Update. J. Cell Sci. 128, 839–852. 10.1242/jcs.161653 25663697 PMC4342575

[B18] Denis K. B. Cabe J. I. Danielsson B. E. Tieu K. V. Mayer C. R. Conway D. E. (2021). The LINC Complex Is Required for Endothelial Cell Adhesion and Adaptation to Shear Stress and Cyclic Stretch. MBoC 32, 1654–1663. 10.1091/mbc.e20-11-0698 34191529 PMC8684736

[B19] Dupont S. Morsut L. Aragona M. Enzo E. Giulitti S. Cordenonsi M. (2011). Role of YAP/TAZ in Mechanotransduction. Nature 474, 179–183. 10.1038/nature10137 21654799

[B20] Ezratty E. J. Bertaux C. Marcantonio E. E. Gundersen G. G. (2009). Clathrin Mediates Integrin Endocytosis for Focal Adhesion Disassembly in Migrating Cells. J. Cell Biol. 187, 733–747. 10.1083/jcb.200904054 19951918 PMC2806590

[B21] Finch-Edmondson M. Sudol M. (2016). Framework to Function: Mechanosensitive Regulators of Gene Transcription. Cell. Mol. Biol. Lett. 21, 28. 10.1186/s11658-016-0028-7 28536630 PMC5415767

[B22] Fracchia A. Asraf T. Salmon-Divon M. Gerlitz G. (2020). Increased Lamin B1 Levels Promote Cell Migration by Altering Perinuclear Actin Organization. Cells 9, 2161. 10.3390/cells9102161 PMC7598699 32987785

[B23] Fukuda S. P. Matsui T. S. Ichikawa T. Furukawa T. Kioka N. Fukushima S. (2017). Cellular Force Assay Detects Altered Contractility Caused by a Nephritis‐associated Mutation in Nonmuscle Myosin IIA. Dev. Growth Differ. 59, 423–433. 10.1111/dgd.12379 28714588

[B24] Gardel M. L. Schneider I. C. Aratyn-Schaus Y. Waterman C. M. (2010). Mechanical Integration of Actin and Adhesion Dynamics in Cell Migration. Annu. Rev. Cell Dev. Biol. 26, 315–333. 10.1146/annurev.cellbio.011209.122036 19575647 PMC4437624

[B25] Hamidi H. Ivaska J. (2018). Every Step of the Way: Integrins in Cancer Progression and Metastasis. Nat. Rev. Cancer. 18, 533–548. 10.1038/s41568-018-0038-z 30002479 PMC6629548

[B26] Hao H. Starr D. A. (2019). SUN/KASH Interactions Facilitate Force Transmission across the Nuclear Envelope. Nucleus 10, 73–80. 10.1080/19491034.2019.1595313 30888237 PMC6527376

[B27] Haque F. Mazzeo D. Patel J. T. Smallwood D. T. Ellis J. A. Shanahan C. M. (2010). Mammalian SUN Protein Interaction Networks at the Inner Nuclear Membrane and Their Role in Laminopathy Disease Processes. J. Biol. Chem. 285, 3487–3498. 10.1074/jbc.m109.071910 19933576 PMC2823409

[B28] Hieda M. (2017). Implications for Diverse Functions of the LINC Complexes Based on the Structure. Cells 6, 3. 10.3390/cells6010003 PMC5371868 28134781

[B29] Hieda M. Isokane M. Koizumi M. Higashi C. Tachibana T. Shudou M. (2008). Membrane-anchored Growth Factor, HB-EGF, on the Cell Surface Targeted to the Inner Nuclear Membrane. J. Cell Biol. 180, 763–769. 10.1083/jcb.200710022 18299347 PMC2373455

[B30] Hieda M. Matsumoto T. Isobe M. Kurono S. Yuka K. Kametaka S. (2021). The Sun2-Nesprin-2 LINC Complex and KIF20A Function in the Golgi Dispersal. Sci. Rep. 11, 5358. 10.1038/s41598-021-84750-4 33686165 PMC7940470

[B31] Hieda M. Matsuura N. Kimura H. (2015). Histone Modifications Associated with Cancer Cell Migration and Invasion. Methods Mol. Biol. 1238, 301–317. 10.1007/978-1-4939-1804-1_16 25421667

[B32] Horn H. F. (2014). LINC Complex Proteins in Development and Disease. Curr. Top. Dev. Biol. 109, 287–321. 10.1016/b978-0-12-397920-9.00004-4 24947240

[B33] Horton E. R. Humphries J. D. Stutchbury B. Jacquemet G. Ballestrem C. Barry S. T. (2016). Modulation of FAK and Src Adhesion Signaling Occurs Independently of Adhesion Complex Composition. J. Cell Biol. 212, 349–364. 10.1083/jcb.201508080 26833789 PMC4739608

[B34] Ichikawa T. Kita M. Matsui T. S. Nagasato A. I. Araki T. Chiang S. H. (2017). Vinexin Family (SORBS) Proteins Play Different Roles in Stiffness-Sensing and Contractile Force Generation. J. Cell Sci. 130, 3517–3531. 10.1242/jcs.200691 28864765 PMC5665443

[B35] Imaizumi H. Sato K. Nishihara A. Minami K. Koizumi M. Matsuura N. (2018). X‐ray‐enhanced Cancer Cell Migration Requires the Linker of Nucleoskeleton and Cytoskeleton Complex. Cancer Sci. 109, 1158–1165. 10.1111/cas.13545 29465769 PMC5891189

[B36] Iyer K. V. Pulford S. Mogilner A. Shivashankar G. V. (2012). Mechanical Activation of Cells Induces Chromatin Remodeling Preceding MKL Nuclear Transport. Biophysical J. 103, 1416–1428. 10.1016/j.bpj.2012.08.041 PMC3471483 23062334

[B37] Kang N. Matsui T. S. Liu S. Fujiwara S. Deguchi S. (2020). Comprehensive Analysis on the Whole Rho‐GAP Family Reveals that ARHGAP4 Suppresses EMT in Epithelial Cells under Negative Regulation by Septin9. FASEB J. 34, 8326–8340. 10.1096/fj.201902750rr 32378260

[B38] Kato T. Enomoto A. Watanabe T. Haga H. Ishida S. Kondo Y. (2014). TRIM27/MRTF-B-dependent Integrin β1 Expression Defines Leading Cells in Cancer Cell Collectives. Cell Rep. 7, 1156–1167. 10.1016/j.celrep.2014.03.068 24794433

[B39] Ketema M. Wilhelmsen K. Kuikman I. Janssen H. Hodzic D. Sonnenberg A. (2007). Requirements for the Localization of Nesprin-3 at the Nuclear Envelope and its Interaction with Plectin. J. Cell Sci. 120 (Pt 19), 3384–3394. 10.1242/jcs.014191 17881500

[B40] Khan R. B. Goult B. T. (2019). Adhesions Assemble!-Autoinhibition as a Major Regulatory Mechanism of Integrin-Mediated Adhesion. Front. Mol. Biosci. 6, 144. 10.3389/fmolb.2019.00144 31921890 PMC6927945

[B41] Kuo J.-C. Han X. Hsiao C.-T. Yates III J. R. 3rd Waterman C. M. (2011). Analysis of the Myosin-II-Responsive Focal Adhesion Proteome Reveals a Role for β-Pix in Negative Regulation of Focal Adhesion Maturation. Nat. Cell Biol. 13, 383–393. 10.1038/ncb2216 21423176 PMC3279191

[B42] Lee S.-w. Otto J. J. (1997). Vinculin and Talin: Kinetics of Entry and Exit from the Cytoskeletal Pool. Cell Motil. Cytoskelet. 36, 101–111. 10.1002/(sici)1097-0169(1997)36:2<101:aid-cm1>3.0.co;2-c 9015199

[B43] Lobert V. H. Brech A. Pedersen N. M. Wesche J. Oppelt A. Malerød L. (2010). Ubiquitination of α5β1 Integrin Controls Fibroblast Migration through Lysosomal Degradation of Fibronectin-Integrin Complexes. Dev. Cell 19, 148–159. 10.1016/j.devcel.2010.06.010 20643357

[B44] Lock J. G. Strömblad B. S. (2008). Cell-matrix Adhesion Complexes: Master Control Machinery of Cell Migration. Semin. Cancer Biol. 18, 65–76. 10.1016/j.semcancer.2007.10.001 18023204

[B45] Lombardi M. L. Jaalouk D. E. Shanahan C. M. Burke B. Roux K. J. Lammerding J. (2011). The Interaction between Nesprins and Sun Proteins at the Nuclear Envelope Is Critical for Force Transmission between the Nucleus and Cytoskeleton. J. Biol. Chem. 286, 26743–26753. 10.1074/jbc.M111.233700 21652697 PMC3143636

[B46] Lovett D. B. Shekhar N. Nickerson J. A. Roux K. J. Lele T. P. (2013). Modulation of Nuclear Shape by Substrate Rigidity. Cell. Mol. Bioeng. 6, 230–238. 10.1007/s12195-013-0270-2 23914256 PMC3727663

[B47] Luque A. Gómez M. Puzon W. Takada Y. Sánchez-Madrid F. Cabañas C. (1996). Activated Conformations of Very Late Activation Integrins Detected by a Group of Antibodies (HUTS) Specific for a Novel Regulatory Region (355–425) of the Common Beta 1 Chain. J. Biol. Chem. 271, 11067–11075. 10.1074/jbc.271.19.11067 8626649

[B48] Luxton G. W. G. Gomes E. R. Folker E. S. Vintinner E. Gundersen G. G. (2010). Linear Arrays of Nuclear Envelope Proteins Harness Retrograde Actin Flow for Nuclear Movement. Science 329 (5994), 956–959. 10.1126/science.1189072 20724637 PMC3938394

[B49] Maekawa M. Tanigawa K. Sakaue T. Hiyoshi H. Kubota E. Joh T. (2017). Cullin-3 and its Adaptor Protein ANKFY1 Determine the Surface Level of Integrin β1 in Endothelial Cells. Biol. Open. 6, 1707–1719. 10.1242/bio.029579 29038302 PMC5703617

[B50] Matsumoto A. Hieda M. Yokoyama Y. Nishioka Y. Yoshidome K. Tsujimoto M. (2015). Global Loss of a Nuclear Lamina Component, Lamin A/C, and LINC Complex Components SUN1, SUN2, and Nesprin-2 in Breast Cancer. Cancer Med. 4, 1547–1557. 10.1002/cam4.495 26175118 PMC4618625

[B51] May C. K. Carroll C. W. (2018). Differential Incorporation of SUN-Domain Proteins into LINC Complexes Is Coupled to Gene Expression. PLOS ONE 13, e0197621. 10.1371/journal.pone.0197621 29813079 PMC5973619

[B52] Meinke P. Schirmer E. C. (2015). LINC’ing Form and Function at the Nuclear Envelope. FEBS Lett. 589 (19 Pt A), 2514–2521. 10.1016/j.febslet.2015.06.011 26096784

[B53] Miano J. M. Long X. Fujiwara K. (2007). Serum Response Factor: Master Regulator of the Actin Cytoskeleton and Contractile Apparatus. Am. J. Physiol. Cell Physiol. 292, C70–C81. 10.1152/ajpcell.00386.2006 16928770

[B54] Nader G. P. Ezratty E. J. Gundersen G. G. (2016). FAK, Talin and PIPKIγ Regulate Endocytosed Integrin Activation to Polarize Focal Adhesion Assembly. Nat. Cell Biol. 18, 491–503. 10.1038/ncb3333 27043085

[B55] Nishioka Y. Imaizumi H. Imada J. Katahira J. Matsuura N. Hieda M. (2016). Sun1 Splice Variants, SUN1_888, SUN. Nucleus. 7. 1_785, and Predominant SUN1_916, Variably Function in Directional Cell Migration. Nucleus 7, 572–584. 10.1080/19491034.2016.1260802 27858498 PMC5214592

[B56] Oakes P. W. Gardel M. L. (2014). Stressing the Limits of Focal Adhesion Mechanosensitivity. Curr. Opin. Cell Biol. 30, 68–73. 10.1016/j.ceb.2014.06.003 24998185 PMC4459577

[B57] Omachi T. Ichikawa T. Kimura Y. Ueda K. Kioka N. (2017). Vinculin Association with Actin Cytoskeleton Is Necessary for Stiffness-dependent Regulation of Vinculin Behavior. PLOS ONE 12, e0175324. 10.1371/journal.pone.0175324 28388663 PMC5384775

[B58] Ostlund C. Folker E. S. Choi J. C. Gomes E. R. Gundersen G. G. Worman H. J. (2009). Dynamics and Molecular Interactions of Linker of Nucleoskeleton and Cytoskeleton (LINC) Complex Proteins. J. Cell Sci. 122, 4099–4108. 10.1242/jcs.057075 19843581 PMC2776502

[B59] Padmakumar V. C Abraham S. Braune S. Noegel A. A. Tunggal B. Karakesisoglou I. (2004). Enaptin, a Giant Actin-Binding Protein, Is an Element of the Nuclear Membrane and the Actin Cytoskeleton. Exp. Cell Res. 295, 330–339. 10.1016/j.yexcr.2004.01.014 15093733

[B60] Padmakumar V. C. Libotte T. Lu W. Zaim H. Abraham S. Noegel A. A. (2005). The Inner Nuclear Membrane Protein Sun1 Mediates the Anchorage of Nesprin-2 to the Nuclear Envelope. J. Cell Sci. 118 (Pt 15), 3419–3430. 10.1242/jcs.02471 16079285

[B61] Panciera T. Azzolin L. Cordenonsi M. Piccolo S. (2017). Mechanobiology of YAP and TAZ in Physiology and Disease. Nat. Rev. Mol. Cell Biol. 18, 758–770. 10.1038/nrm.2017.87 28951564 PMC6192510

[B62] Pasapera A. M. Schneider I. C. Rericha E. Schlaepfer D. D. Waterman C. M. (2010). Myosin II Activity Regulates Vinculin Recruitment to Focal Adhesions through FAK-Mediated Paxillin Phosphorylation. J. Cell Biol. 188, 877–890. 10.1083/jcb.200906012 20308429 PMC2845065

[B63] Poh Y. C. Shevtsov S. P. Chowdhury F. Wu D. C. Na S. Dundr M. (2012). Dynamic Force-Induced Direct Dissociation of Protein Complexes in a Nuclear Body in Living Cells. Nat. Commun. 3, 866. 10.1038/ncomms1873 22643893 PMC3388544

[B64] Rashmi R. N. Eckes B. Glöckner G. Groth M. Neumann S. Gloy J. (2012). The Nuclear Envelope Protein Nesprin-2 Has Roles in Cell Proliferation and Differentiation during Wound Healing. Nucleus 3, 172–186. 10.4161/nucl.19090 22198684 PMC3383573

[B65] Razafsky D. Hodzic D. (2009). Bringing KASH under the SUN: the Many Faces of Nucleo-Cytoskeletal Connections. J. Cell Biol. 186, 461–472. 10.1083/jcb.200906068 19687252 PMC2733748

[B66] Sanchez-Madrid F. Krensky A. M. Ware C. F. Robbins E. Strominger J. L. Burakoff S. J. (1982). Three Distinct Antigens Associated with Human T-Lymphocyte-Mediated Cytolysis: LFA-1, LFA-2, and LFA-3. Proc. Natl. Acad. Sci. U. S. A. 79, 7489–7493. 10.1073/pnas.79.23.7489 6984191 PMC347365

[B67] Satomi E. Ueda M. Katahira J. Hieda M. (2020). The SUN1 Splicing Variants SUN1_888 and SUN1_916 Differentially Regulate Nucleolar Structure. Genes cells. 25, 730–740. 10.1111/gtc.12807 32931086

[B68] Sawada Y. Sheetz M. P. (2002). Force Transduction by Triton Cytoskeletons. J. Cell Biol. 156, 609–615. 10.1083/jcb.200110068 11839769 PMC2174068

[B69] Schiller H. B. Hermann M. R. Polleux J. Vignaud T. Zanivan S. Friedel C. C. (2013). β1- and αv-class Integrins Cooperate to Regulate Myosin II during Rigidity Sensing of Fibronectin-Based Microenvironments. Nat. Cell Biol. 15, 625–636. 10.1038/ncb2747 23708002

[B70] Schlaepfer D. D. Hauck C. R. Sieg D. J. (1999). Signaling through Focal Adhesion Kinase. Prog. Biophys. Mol. Biol. 71, 435–478. 10.1016/s0079-6107(98)00052-2 10354709

[B71] Sharma V. P. Williams J. Leung E. Sanders J. Eddy R. Castracane J. (2021). SUN-MKL1 Crosstalk Regulates Nuclear Deformation and Fast Motility of Breast Carcinoma Cells in Fibrillar ECM Microenvironment. Cells 10, 1549. 10.3390/cells10061549 34205257 PMC8234170

[B72] Shattil S. J. Kim C. Ginsberg M. H. (2010). The Final Steps of Integrin Activation: the End Game. Nat. Rev. Mol. Cell Biol. 11, 288–300. 10.1038/nrm2871 20308986 PMC3929966

[B73] Simon D. N. Wilson K. L. (2011). The Nucleoskeleton as a Genome-Associated Dynamic “Network of Networks”. Nat. Rev. Mol. Cell Biol. 12, 695–708. 10.1038/nrm3207 21971041

[B92] Sordat I. Decraene C. Silvestre T. Petermann O. Auffray C. Piétu G. (2003). Complementary DNA Arrays Identify CD63 Tetraspanin and Alpha3 Integrin Chain as Differentially Expressed in Low and High Metastatic Human Colon Carcinoma Cells. Lab. Invest. 82, 1715–1724. 10.1097/01.lab.0000044350.18215.0d 12480921

[B74] Sosa B. A. Rothballer A. Kutay U. Schwartz T. U. (2012). LINC Complexes Form by Binding of Three KASH Peptides to Domain Interfaces of Trimeric SUN Proteins. Cell 149, 1035–1047. 10.1016/j.cell.2012.03.046 22632968 PMC3383001

[B75] Starr D. A. (2011). KASH and SUN Proteins. Curr. Biol. 21, R414–R415. 10.1016/j.cub.2011.04.022 21640895 PMC5518751

[B76] Steinberg F. Heesom K. J. Bass M. D. Cullen P. J. (2012). SNX17 Protects Integrins from Degradation by Sorting between Lysosomal and Recycling Pathways. J. Cell Biol. 197, 219–230. 10.1083/jcb.201111121 22492727 PMC3328392

[B77] Stewart-Hutchinson P. J. Hale C. M. Wirtz D. Hodzic D. (2008). Structural Requirements for the Assembly of LINC Complexes and Their Function in Cellular Mechanical Stiffness. Exp. Cell Res. 314, 1892–1905. 10.1016/j.yexcr.2008.02.022 18396275 PMC2562747

[B78] Thakar K. May C. K. Rogers A. Carroll C. W. (2017). Opposing Roles for Distinct LINC Complexes in Regulation of the Small GTPase RhoA. Mol. Biol. Cell. 28, 182–191. 10.1091/mbc.e16-06-0467 28035049 PMC5221622

[B79] Toh K. C. Ramdas N. M. Shivashankar G. V. (2015). Actin Cytoskeleton Differentially Alters the Dynamics of Lamin A, HP1α and H2B Core Histone Proteins to Remodel Chromatin Condensation State in Living Cells. Integr. Biol. 7, 1309–1317. 10.1039/c5ib00027k 26359759

[B91] Tsuji T. Kawada Y. Kai-Murozono M. Komatsu S. Han S. A. Takeuchi K. (2002). Regulation of melanoma cell migration and invasion by laminin-5 and alpha3beta1 integrin (VLA-3). Clin. Exp. Metastasis 19, 127–134. 10.1023/a:1014573204062 11964076

[B80] Versaevel M. Grevesse T. Gabriele S. (2012). Spatial Coordination between Cell and Nuclear Shape within Micropatterned Endothelial Cells. Nat. Commun. 3, 671. 10.1038/ncomms1668 22334074

[B81] Wang J. Y. Yu I. S. Huang C. C. Chen C. Y. Wang W. P. Lin S. W. (2015). Sun1 Deficiency Leads to Cerebellar Ataxia in Mice. Dis. Model Mech. 8, 957–967. 10.1242/dmm.019240 26035387 PMC4527285

[B82] Wong X. Loo T. H. Stewart C. L. (2021). LINC Complex Regulation of Genome Organization and Function. Curr. Opin. Genet. Dev. 67, 130–141. 10.1016/j.gde.2020.12.007 33524904

[B83] Woychek A. Jones J. C. R. (2019). Nesprin-2G Knockout Fibroblasts Exhibit Reduced Migration, Changes in Focal Adhesion Composition, and Reduced Ability to Generate Traction Forces. Cytoskelet. Hob. 76, 200–208. 10.1002/cm.21515 30667166

[B84] Yamashita H. Ichikawa T. Matsuyama D. Kimura Y. Ueda K. Craig S. W. (2014). The Role of the Interaction of the Vinculin Proline-Rich Linker Region with Vinexin α in Sensing the Stiffness of the Extracellular Matrix. J. Cell Sci. 127, 1875–1886. 10.1242/jcs.133645 24554436

[B85] Yokoyama Y. Matsumoto A. Hieda M. Shinchi Y. Ogihara E. Hamada M. (2014). Loss of Histone H4K20 Trimethylation Predicts Poor Prognosis in Breast Cancer and Is Associated with Invasive Activity. Breast Cancer Res. 16, R66. 10.1186/bcr3681 24953066 PMC4229880

[B90] Yoshimasu T. Sakurai T. Oura S. Hirai I. Tanino H. Kokawa Y. (2004). Increased Expression of Integrin Alpha3beta1 in Highly Brain Metastatic Subclone of a Human Non-Small Cell Lung Cancer Cell Line. Cancer Sci. 95, 142–148. 10.1111/j.1349-7006.2004.tb03195.x 14965364 PMC11158200

[B86] Zaidel-Bar R. Ballestrem C. Kam Z. Geiger B. (2003). Early Molecular Events in the Assembly of Matrix Adhesions at the Leading Edge of Migrating Cells. J. Cell Sci. 116, 4605–4613. 10.1242/jcs.00792 14576354

[B87] Zhang Q. Kota K. P. Alam S. G. Nickerson J. A. Dickinson R. B. Lele T. P. (2016). Coordinated Dynamics of RNA Splicing Speckles in the Nucleus. J. Cell. Physiol. 231, 1269–1275. 10.1002/jcp.25224 26496460 PMC4755833

[B88] Zhen Y. Y. Libotte T. Munck M. Noegel A. A. Korenbaum E. (2002). NUANCE, a Giant Protein Connecting the Nucleus and Actin Cytoskeleton. J. Cell Sci. 115 (Pt 15), 3207–3222. 10.1242/jcs.115.15.3207 12118075

[B89] Zhu R. Antoku S. Gundersen G. G. (2017). Centrifugal Displacement of Nuclei Reveals Multiple LINC Complex Mechanisms for Homeostatic Nuclear Positioning. Curr. Biol. 27, 3097–3110. e5. 10.1016/j.cub.2017.08.073 28988861 PMC5688853

