# Supplementary material for: Inner Nuclear Membrane Protein, SUN1, is Required for Cytoskeletal Force Generation and Focal Adhesion Maturation
Source: Front Cell Dev Biol. 2022 May 18;10:885859. doi: 10.3389/fcell.2022.885859 (PMC9157646; doi:10.3389/fcell.2022.885859)

## Supplementary Materials and Methods

### Materials

Rabbit anti-Myc pAb was purchased from Medical & Biological Laboratories (Tokyo, Japan). Rabbit anti vinculin mAb (clone E1E9V) was purchased from Cell Signaling (Danvers, MA). Myc-tagged mouse SUN1 is described previously (Hieda et al., 2021).

### Focal adhesion disassembly assay

A microtubule-induced focal adhesion disassembly experiment was performed as previously described (Ezratty et al., 2005). In brief, cells were treated with 10 μM nocodazole for 4 h to completely depolymerize microtubules. The drug was washed out with serum-free medium, and microtubules were allowed to repolymerize for the indicated period.

### Measurement of GTP-RhoA

The GTP-RhoA level was measured using a G-LISA RhoA activation Assay Biochem Kit (Cytoskeleton Inc.) according to the manufacturer's instructions.

### Supplementary Figure Caption

**Supplementary Figure 1. Actin fiber levels and organization are disrupted in the SUN1-depleted cells.**

**(A).** Low magnification (×20) image of actin staining in the control or SUN1-depleted cells. Arrows show the accumulation of actin signal at the edge of the cells. Scale bar, 20 μm. **(B).** Cells were transfected with siSUN1. After 24 h, the cells were transfected with Myc-tagged mouse SUN1 and incubated for 24 h. The cells were then stained with anti-Myc mAb and stained with rhodamine–phalloidin. Arrows show Myc-tagged mouse SUN1-transfected cells. Other cells on the right-hand side were not transfected. Scale bar, 10 μm. **(C).** Confocal images of actin staining. Arrows show sub-nuclear actin structures. **(D).** The values represent the mean of the relative intensity of β-actin expression to β-tubulin in the western blotting ± standard deviation (SD). **(E).** GTP-RhoA level in the lysate of siNC- or siSUN1 transfected cells was measured as absorbance of 490 nm -transfected cells. **(F).**

**Supplementary Figure 2. SUN1-depleted cells have the ability to turn over their focal adhesions**

**(A).** A microtubule-induced focal adhesion disassembly experiment (Ezratty et al., 2005) was performed using siNC- or siSUN1-transfected cells. **(B).** Cells were transfected with siSUN1. After 24 h, the cells were transfected with Myc-tagged mouse SUN1 and incubated for 24 h. The cells were treated with 0.5% Triton X-100 for 5 min on ice and fixed. The cells were then stained with anti-vinculin mAb and anti-Myc pAb. An arrow and an asterisk show Myc-tagged mouse SUN1-transfected and -untransfected cells, respectively. Scale bar, 10 μm. **(C).** The values represent the mean of the relative intensity of vinculin expression to β-tubulin in the western blotting ± standard deviation (SD).

**Supplementary Figure 3. SUN1 depletion affects expression of integrin β1**

**(A).** Total integrin β1 staining pattern in siNC or siSUN1 transfected MCF20A cells. Scale bar, 10 μm. **(B).** Cells were treated with 0.5% Triton X-100 for 5 min on ice. The cells were then fixed and stained with rabbit anti-vinculin mAb and mouse anti-active integrin β1 mAb (12G10). Scale bar, 10 μm. **(C).** Cells were transfected with siSUN1. After 24 h, the cells were transfected with Myc-tagged mouse SUN1 and incubated for 24 h. The cells were then stained with anti-Myc pAb and anti-active integrin β1 mAb (12G10) or anti-zyxin mAb. Scale bar, 10 μm.

**Supplementary Figure 4. Integrin β1 staining during internalization and recycling assays**

**(A).** Cells were transfected with siSUN1 or siNC. After 48 h of incubation, cell surface integrin β1 was labeled with Alexa 488-conjugated TS2/16 mAb (time: 0 min) and chased for 10 min (time: 10 min). **(H).** Cells were treated with siSUN1 or siNC. After 48 h of incubation, cell surface integrin β1 was labeled with Alexa 488-conjugated TS2/16 mAb and chased for 60 min to allow endocytosis. Afterward, the remaining fluorescence at the cell surface was quenched (time: 0 min), and cells were incubated to allow trafficking from the endosomes to the plasma membrane for the indicated time. Next, cell surface fluorescence was again quenched.

**Supplementary Figure 5. Area of cell spreading of the SUN1-knocked out HeLa cells**

The area of cell spreading was quantified using ImageJ software. The values represent the relative area of cell spreading to the parental cells ± standard error of the mean (SEM). ****P* < 0.005, **P* < 0.05 compared with the parental HeLa cells

Ueda et al., Figure S1
Actin fiber level and organization are disrupted in SUN1-depleted cells

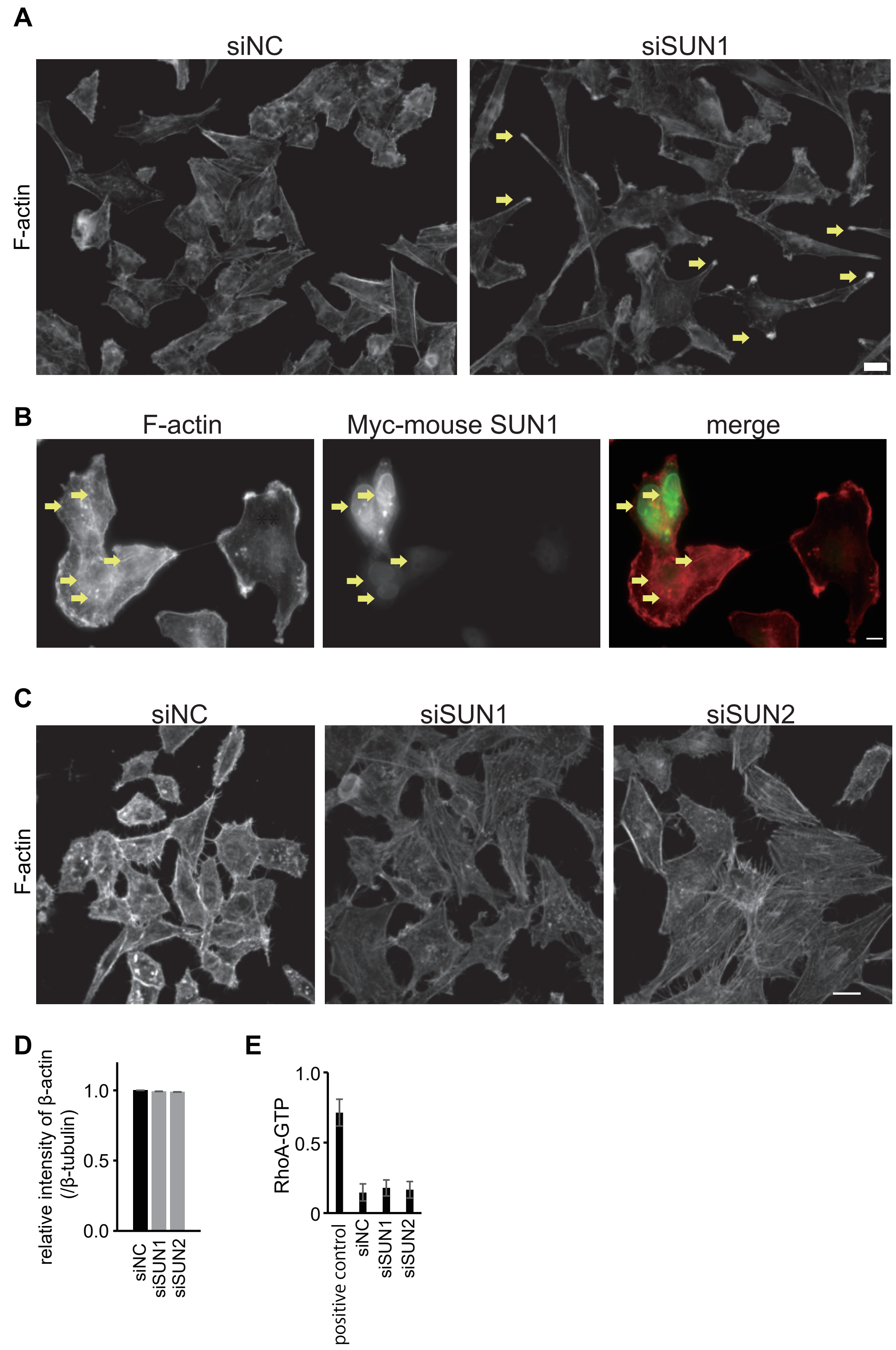

Ueda et al., Fig. S2

SUN1 depleted cells has ability to turn over their focal adhesions

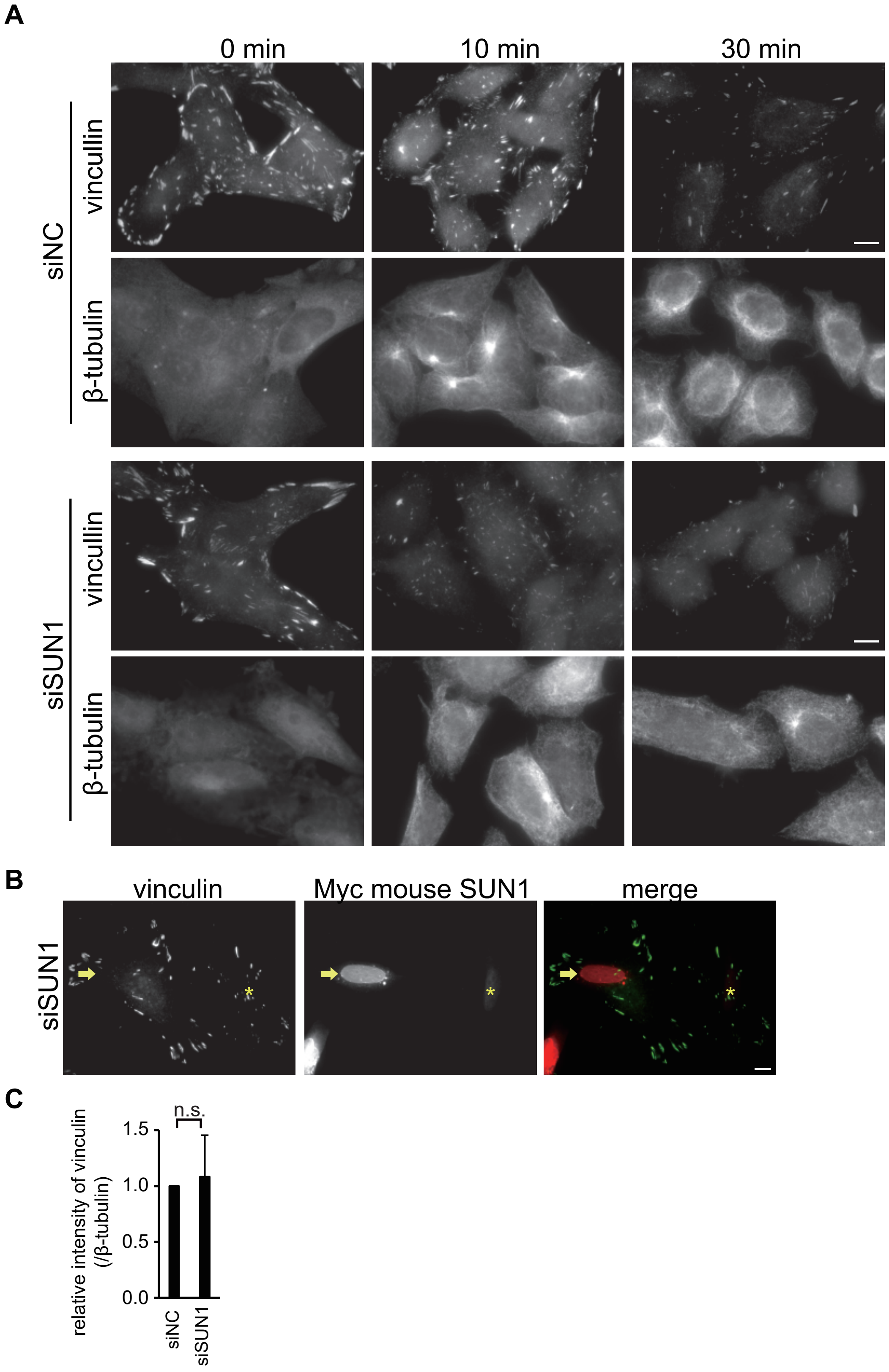

Ueda et al., Figure S3
SUN1 depletion affects integrin β1 expression

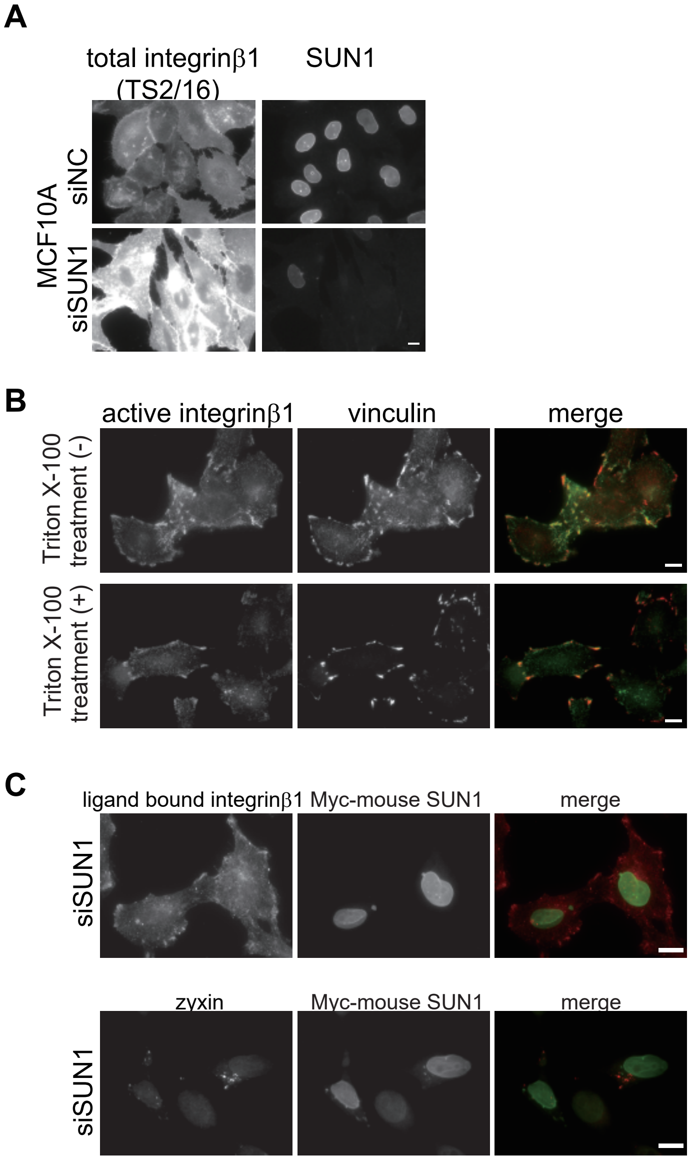

Ueda et al., Figure S4

Integrin β1 staining during internalization and recycling assays

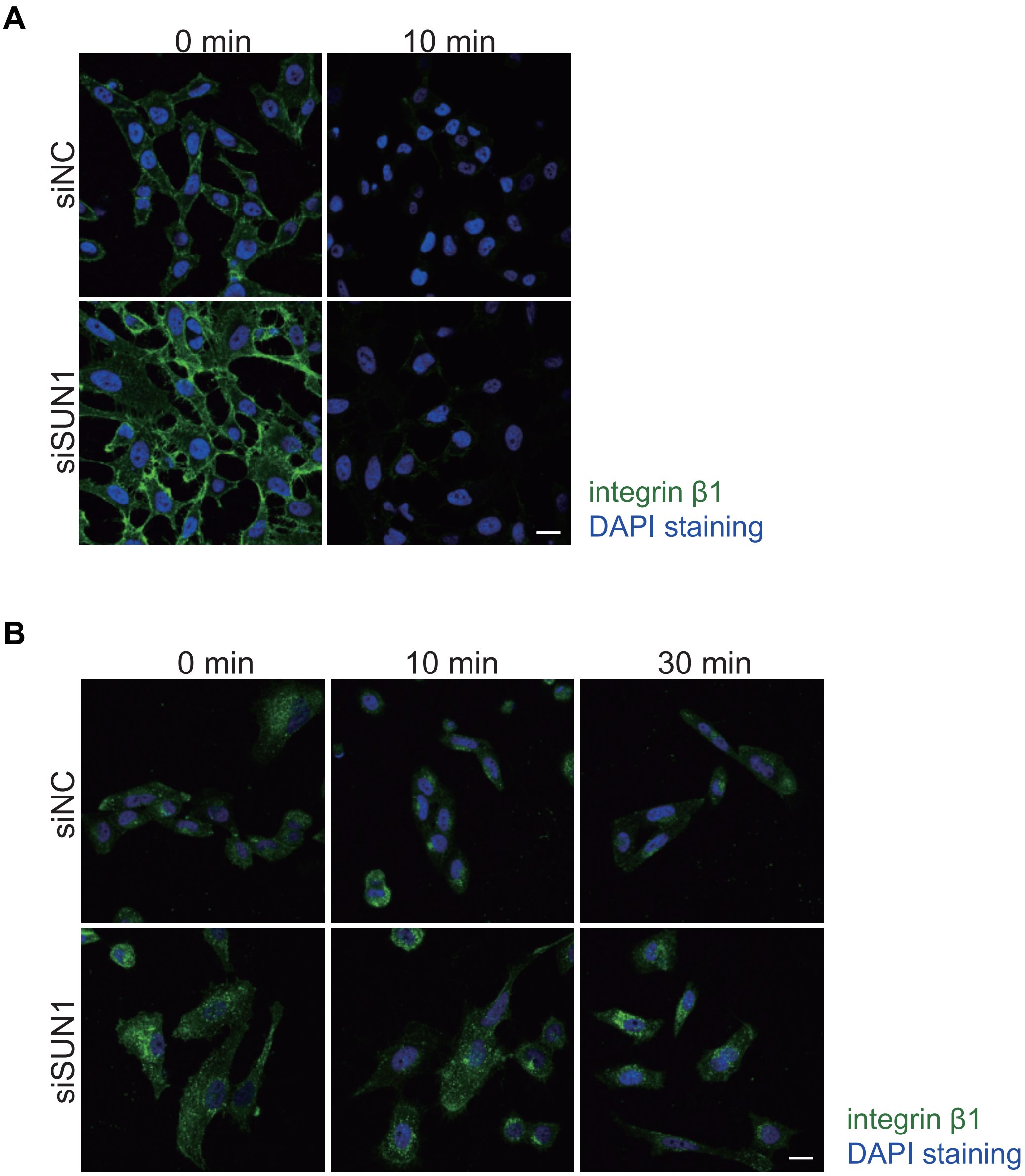

Ueda et al., Figure S5
Cell spreading area of SUN1 knock out cells

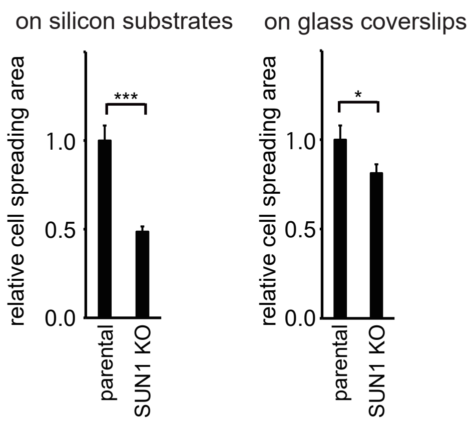

Supplement: Supplementary file 1 [file Presentation1.pdf]
